# microRNA-mediated differential expression of TRMU, GTPBP3 and MTO1 in cell models of mitochondrial-DNA diseases

**DOI:** 10.1038/s41598-017-06553-w

**Published:** 2017-07-24

**Authors:** Salvador Meseguer, Olga Boix, Carmen Navarro-González, Magda Villarroya, Rachid Boutoual, Sonia Emperador, Elena García-Arumí, Julio Montoya, M.-Eugenia Armengod

**Affiliations:** 1 Laboratory of RNA Modification and Mitochondrial Diseases, Centro de Investigación Príncipe Felipe, Valencia, Spain; 2 Hospital Universitario Vall d’Hebron (Barcelona, Spain) and Biomedical Research Networking Centre for Rare Diseases CIBERER, node 701, Barcelona, Spain; 3 Universidad de Zaragoza - CIBERER (node 727)-Instituto de Investigación Sanitaria de Aragón, Zaragoza, Spain; 4 CIBERER node 721, Valencia, Spain

## Abstract

Mitochondrial diseases due to mutations in the mitochondrial (mt) DNA are heterogeneous in clinical manifestations but usually include OXPHOS dysfunction. Mechanisms by which OXPHOS dysfunction contributes to the disease phenotype invoke, apart from cell energy deficit, maladaptive responses to mitochondria-to-nucleus retrograde signaling. Here we used five different cybrid models of mtDNA diseases to demonstrate that the expression of the nuclear-encoded mt-tRNA modification enzymes TRMU, GTPBP3 and MTO1 varies in response to specific pathological mtDNA mutations, thus altering the modification status of mt-tRNAs. Importantly, we demonstrated that the expression of TRMU, GTPBP3 and MTO1 is regulated by different miRNAs, which are induced by retrograde signals like ROS and Ca^2+^ via different pathways. Our data suggest that the up- or down-regulation of the mt-tRNA modification enzymes is part of a cellular response to cope with a stoichiometric imbalance between mtDNA- and nuclear-encoded OXPHOS subunits. However, this miRNA-mediated response fails to provide full protection from the OXPHOS dysfunction; rather, it appears to aggravate the phenotype since transfection of the mutant cybrids with miRNA antagonists improves the energetic state of the cells, which opens up options for new therapeutic approaches.

## Introduction

Mitochondrial diseases caused by impairment of mitochondrial translation are highly heterogeneous in etiology and clinical manifestations but usually include oxidative phosphorylation (OXPHOS) dysfunction^1^. Mechanisms by which OXPHOS dysfunction contributes to the disease phenotype are not well-understood but they likely involve retrograde signaling from mitochondria to nucleus triggered by changes in metabolite homeostasis, such as ROS, Ca^2+^, ADP/ATP and NAD/NADH^2–5^. Understanding how these changes lead to maladaptive responses upon OXPHOS dysfunction may help to find new therapeutic strategies.

The human mitochondrial genome (mtDNA) encodes thirteen structural subunits of the OXPHOS complexes I, III, IV and V, and the 22 tRNAs and 2 rRNAs used for intra-mitochondrial protein synthesis. More than 50% of the pathogenic mtDNA mutations occur in tRNA genes^6^. Some of them affect mt-tRNA^Lys^ and mt-tRNA^Leu^ causing MERRF and MELAS, respectively^7^. These mutations prevent the modification of the anticodon wobble uridine (U34), which disturbs the function of the mutant tRNAs in translation^8, 9^. Modifications of U34 in a mt-tRNA set depend on the nuclear-encoded proteins GTPBP3 and MTO1 (which jointly introduce the taurinomethyl group at position 5 of the pyrimidine ring in mt-tRNA^Lys^, mt-tRNA^Leu^, mt-tRNA^Gln^, mt-tRNA^Glu^, and mt-tRNA^Trp^), and TRMU (which thiolates U34 at position 2 in mt-tRNA^Lys^, mt-tRNA^Gln^, and mt-tRNA^Glu^)^10, 11^. Mutations directly affecting GTPBP3 and MTO1 cause infantile hypertrophic cardiomyopathy^12–14^, whereas TRMU mutations cause infantile hepatopathy, which is fatal in some instances and reversible in others for unknown reasons^15–17^.

Cells carrying MELAS mutations exhibit mitochondrial translation defects, oxidative stress, and diminished respiratory enzyme activity and oxygen consumption^18–21^. In addition to OXPHOS dysfunction resulting from altered mitochondrial translation, other mechanisms contribute to the MELAS phenotype. Thus, the MELAS mutation A3243G induces, in a cell model, a retrograde signaling pathway involving ROS, kinase JNK, retinoid X receptor α and transcriptional coactivator PGC1α^3^. This pathway leads to a decrease in the mRNA levels of nuclear-encoded OXPHOS subunits, thereby aggravating the OXPHOS dysfunction. Moreover, we have recently shown that the high levels of ROS caused by the MELAS mutation A3243G induce, via an NFkB pathway, the expression of microRNA 9/9* (miR-9/9*), which reduces the steady-state levels of TRMU, GTPBP3 and MTO1 because of their mRNAs are direct targets of miR-9 (TRMU and GTPBP3) and miR-9* (MTO1)^4^. Down-regulation of these enzymes affects the U34 modification of non-mutant mt-tRNAs and contributes to the MELAS phenotype in a cell model. These data provided the first evidence that the modification status of mt-tRNAs is dynamic, as previously observed in cytosolic tRNAs^22^, and that cells respond to oxidative stress by reducing the expression of mt-tRNA modification enzymes through the action of a miRNA.

In this paper, we investigate whether deregulation of TRMU, GTPBP3, and MTO1 also participates in the cell response to the stress caused by pathogenic mutations in other mtDNA genes, including non-substrate mt-tRNA and protein-encoding genes. Transmitochondrial cytoplasmic hybrids (cybrids) are appropriate cell models to compare in the same nuclear background the effects of different homoplasmic mtDNA mutations. Thus, we compared the expression of GTPBP3, MTO1 and TRMU genes among cybrid cells carrying mutations in mt-tRNA^Leu^ (m.3243 A > G, MELAS), mt-tRNA^Lys^ (m.8344 A > G, MERRF), mt-tRNA^Trp^ (m.5514 A > G, m.Trp) or mt-tRNA^Val^ (m.1643A > G, m.Val) genes, all of them associated with severe encephalomyopathic phenotypes (Table 1)^23, 24^. In addition, we included in the study the m.14487 T > C mutation in the *ND6* gene (m.ND6), which is causative of a progressive generalized dystonia and bilateral striatal necrosis (Table 1)^25^

**Table 1:** Brief summary of clinical symptoms associated with each mutation.

Cybrid cell name	MELAS	MERRF	m.Val	m.Trp	m.ND6
tRNA/protein and mutation	tRNA ^Leu^ m.3243 A > G (MELAS)	tRNA ^Lys^ m. 8344 A > G (MERRF)	tRNA ^Val^ m. 1643A > G	tRNA ^Trp^ m. 5514 A > G	ND6 m.14487 T > C
**Organ**	**Clinical features**				
Brain	Stroke-like episodes, typically before age 40 years Encephalopathy with seizures and/or dementia Normal early psychomotor development Recurrent headache	Myoclonus Generalized epilepsy Ataxia Dementia Pyramidal signs	Normal early psychomotor development Epilepsy Brain atrophy Dyskinetic movement Dystonic tetraplegia Loss of oral communication	Reduced spontaneous movements	Progressive generalized dystonia and bilateral striatal necrosis
Nerve	Recurrent vomiting	Peripheral neuropathy	Peripheral neuropathy	Global delay in myelination Recurrent vomiting	
Muscle	Mitochondrial myopathy, evidenced by lactic acidosis and/or ragged red fibers (RRF) on muscle biopsy	Myopathy Ragged-red fibers (RRF) in the muscle biopsy Ophthalmoparesis	Muscle atrophy Lactic acidosis	Muscle atrophy Hypotonia Lactic acidosis.	
Heart		Cardiomyopathy			
Eyes		Optic atrophy Pigmentary retinopathy			
Ears		Sensorineural hearing loss			
Systemic		Exercise intolerance Short stature Multiple lipomas		Low weight and height	
Reference	23	23	24	24	25

, as an example of mtDNA mutation affecting a protein-encoding gene. We found that variations in the steady-state levels of U34 modifying proteins are noticeable in all the selected cellular models of mt-DNA diseases, which suggests that regulation of these enzymes is part of the adaptive/maladaptive response to OXPHOS dysfunction. mtDNA mutations lead to up- or down-regulation of specific U34 modification enzymes depending partially on the cellular miRNA expression pattern, which is associated with the levels of retrograde signals. Our work supports the view that stress-induced reprogramming of mt-tRNA modifications occurs under certain pathological conditions and that miRNAs are key regulators of the mt-tRNA modification enzymes and, consequently, of the mt-tRNA functions.

## Results

### The selected disease cell models exhibit different profiles of proteotoxic stress, OXPHOS dysfunction, and retrograde signals

**Figure 1:**
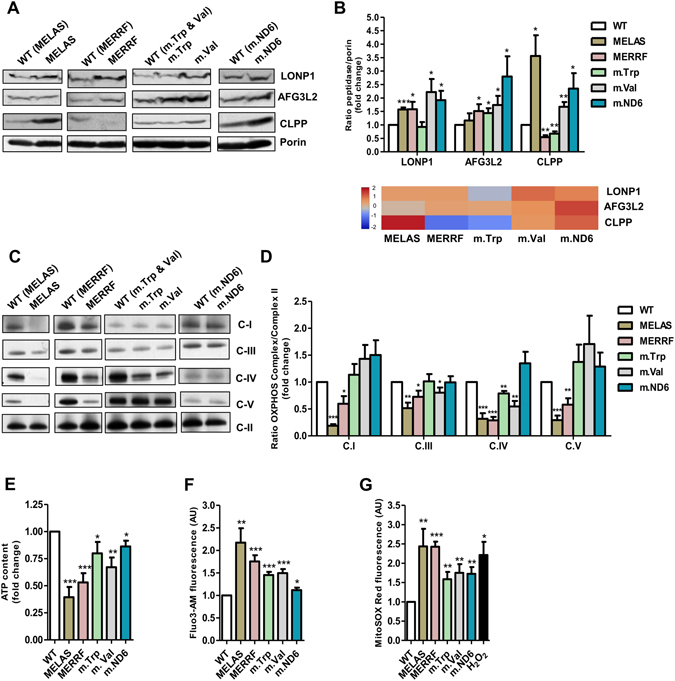
Altered mitochondrial features in cybrid cells carrying MELAS, MERRF, m.5514 A > G (mt-tRNA^Trp^), m.1643A > G (mt-tRNA^Val^), and m.14487 T > C (ND6) mutations. (**A**) Representative western blot of LONP1, AFG3L2 and CLPP peptidases in mutant and wild type (WT) cybrid cells. The membrane was also probed with porin as a loading control. Full-length western blots and lower-exposure blots of porin are included in supplementary information. (**B**) Densitometric analysis of LONP1, AFG3L2 and CLPP normalized to porin and represented as fold change relative to WT (top). Quantitative data are from at least three independent experiments. Results from this analysis are also shown as a heatmap (bottom). The color and the corresponding value in log_2_ scale are depicted on the left. (**C**) Representative Blue Native-PAGE of OXPHOS complexes in mutant and WT cybrid cells. Full-length blots and lower-exposure blots for those with high contrast are included in supplementary information. (**D**) Densitometric analysis of OXPHOS complexes normalized to complex-II (loading control) and represented as fold change relative to WT. (**E**) Cellular ATP determination in mutant and WT cybrid cells. (**F** and **G**) Determination of Ca^2+^ (**F**) and ROS (**G**) by flow cytometry in mutant and WT cybrid cells with Fluo-3 and MitoSOX Red, respectively. All data are the mean ± SEM of at least three different experiments. Differences from WT values were found to be statistically significant at *p < 0.05, **p < 0.01 and ***p < 0.001.

Mutations in mtDNA genes encoding mt-tRNA or OXPHOS subunits may lead to disruption of stoichiometric balance between components of OXPHOS complexes, thus unleashing proteotoxic stress^5, 26, 27^. Common markers of this stress are mitoproteases LONP1, AFG3L2 and CLPP, which, in addition to their roles in protein quality control, are involved in various biological processes such as mtDNA maintenance and mitoribosome assembly^27^. LONP1 is the major matrix protease responsible for degradation of unfolded or misfolded proteins before their aggregation^28^, and also controls mtDNA maintenance and gene expression^27^; AFG3L2 is integrated into the inner mitochondrial membrane and appears to be important for destabilization of OXPHOS complexes containing incorrect proteins, removal of orphaned OXPHOS proteins^26, 29, 30^, ribosome assembly, and synthesis of mtDNA-encoded OXPHOS subunits^31, 32^; finally, CLPP has been recently shown to play an essential role in determining the rate of mitochondrial protein synthesis; loss of CLPP leads to a moderate defect in mitochondrial translation that may have compensatory effects in certain pathogenic contexts^33, 34^. As shown in Fig. 1A and B, the levels of LONP1 were increased in all mutant cybrid lines with exception of m.Trp cells, expression of AFG3L2 was induced in all disease cell models except MELAS, and the levels of CLPP were increased in MELAS, m.Val and m.ND6 cells, whereas they were reduced in MERRF and m.Trp cells. Therefore, there exists a disease-specific expression pattern of these mitoproteases in the cybrid lines. We conclude that each mtDNA mutation triggers a specific protease response as a consequence of a different proteotoxic scenario.

The determination of the steady-state levels of OXPHOS complexes in mutant and wild type (WT) cybrid cells by blue native polyacrylamide gel electrophoresis (BN-PAGE) showed that the severity of OXPHOS defects varies with the mutation, being more drastic in MELAS and MERRF cells, with affectation of complexes I, III, IV and V. Conversely, we could not detect any reduction in the levels of OXPHOS complexes in m.ND6 cells (Fig. 1C and D), despite activity of complex I was found to be decreased in this cybrid line^25^. This finding suggests that complex I may be misfolded and/or misassembled in m.ND6 cells.

It is worthy to mention that the decrease in the steady-state levels of OXPHOS complexes containing mtDNA-encoded subunits (CI, CIII, CIV, and CV) observed in MELAS and MERRF cybrids is not due to a lower content of mtDNA (in relation to nDNA) since no differences in the mtDNA copy number were observed between the mutant and WT cybrid lines (Fig. S1).

The cellular consequences of the OXPHOS dysfunction include changes in a variety of metabolites such as ATP, ROS and Ca^2+^, which act as signals in retrograde mitochondria-nucleus communication. We found 60% and 50% reduction of ATP levels in MELAS and MERRF cybrid cells, respectively, in comparison to WT cybrid cells, whereas the reduction was more moderate in m.Val (~30%), m.Trp (~20%) and m.ND6 cells (~20%) (Fig. 1E). Determination of the intracellular calcium (Ca^2+^) and superoxide anion (O_2_·^−^) levels by flow cytometry showed elevated levels in all the mutant cybrid cells, as compared to WT cells, although these traits were again more prominent in MELAS and MERRF cells (Fig. 1F and G). Notably, the m.ND6 cells exhibited the lowest increase in the intracellular Ca^2+^ levels (Fig. 1F). Altogether these results suggest that the intensity of changes in the retrograde signals ATP, Ca^2+^ and ROS is not equal for all the mutant cybrids, which is likely a consequence of the particular effects of each mtDNA mutation.

### Altered levels of U34 modification enzymes is a common trait in all the selected cybrid lines

**Figure 2:**
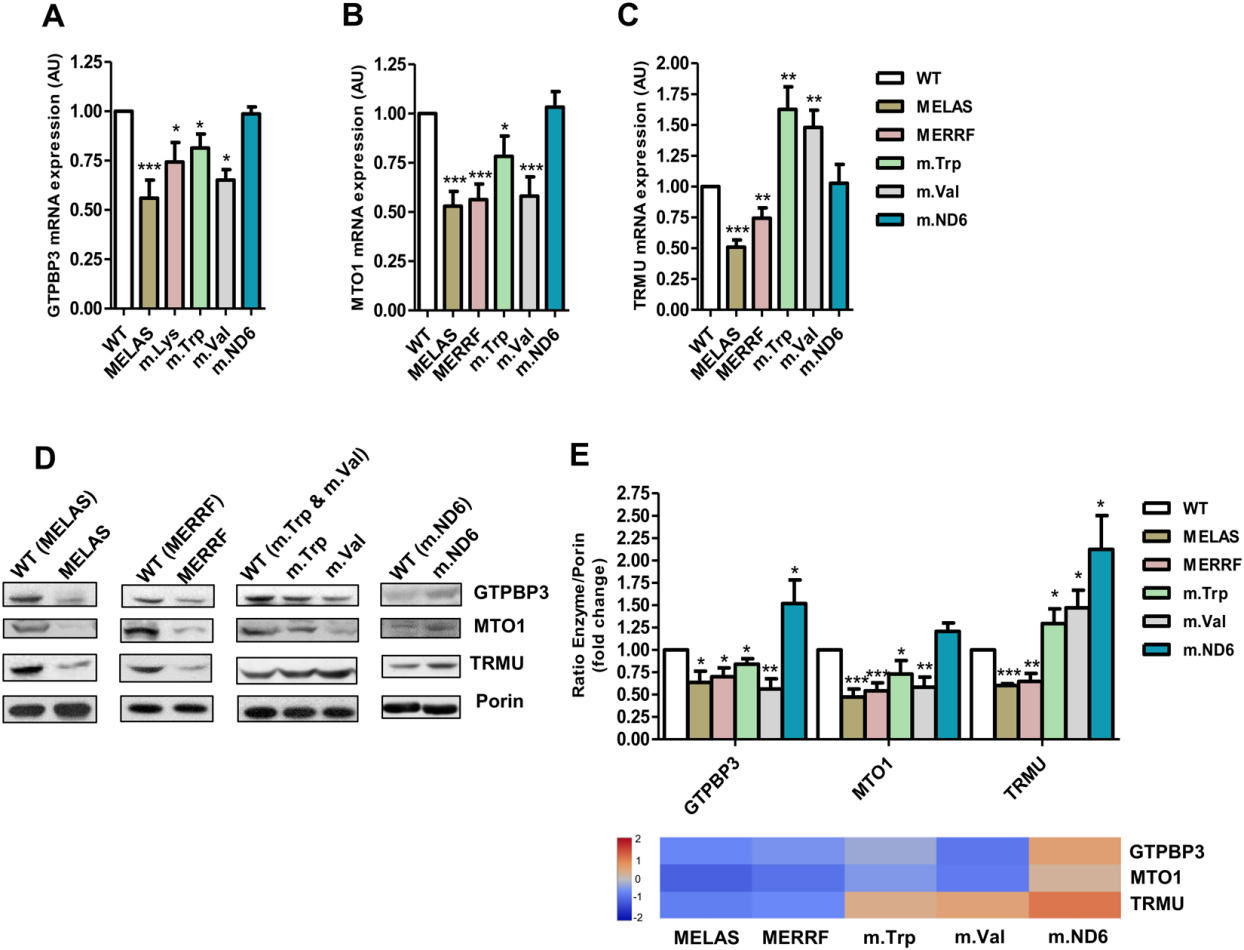
Specific expression pattern of U34 modifying enzymes in the different cybrid cells. (**A–C**) qRT-PCR analysis of *GTPBP3* (**A**), *MTO1* (**B**) and *TRMU* (**C**) mRNA expression in mutant and WT cybrid cells. (**D**) Western blot analysis of GTPBP3, MTO1 and TRMU in mutant and WT cybrid cells. The membrane was also probed with porin as a loading control. Full-length western blots are included in supplementary information. (**E**) Densitometric analysis of GTPBP3, MTO1 and TRMU normalized to porin and represented as fold change relative to WT (top). Results from this analysis are also shown as a heatmap (bottom). The color and the corresponding value in log_2_ scale are depicted on the left. All data are the mean ± SEM of at least three different experiments. Differences from WT values were found to be statistically significant at *p < 0.05, **p < 0.01 and ***p < 0.001. AU: arbitrary units.

The mRNA and protein expression levels of the *GTPBP3*, *MTO1* and *TRMU* genes were evaluated in the cybrid cells by qRT-PCR and Western Blot (Fig. 2). A reduction in the mRNA and protein levels of *GTPBP3* and *MTO1* was found in all the disease cell models except m.ND6 (Fig. 2A–E). The expression of TRMU was decreased in MELAS and MERRF cybrids but increased in m.Trp and m.Val cells (Fig. 2C–E). A tendency for increased levels of the three U34 modification enzymes was observed in m.ND6 cells (Fig. 2E), despite no increase was detected at mRNA levels (Fig. 2A–C).

**Figure 3:**
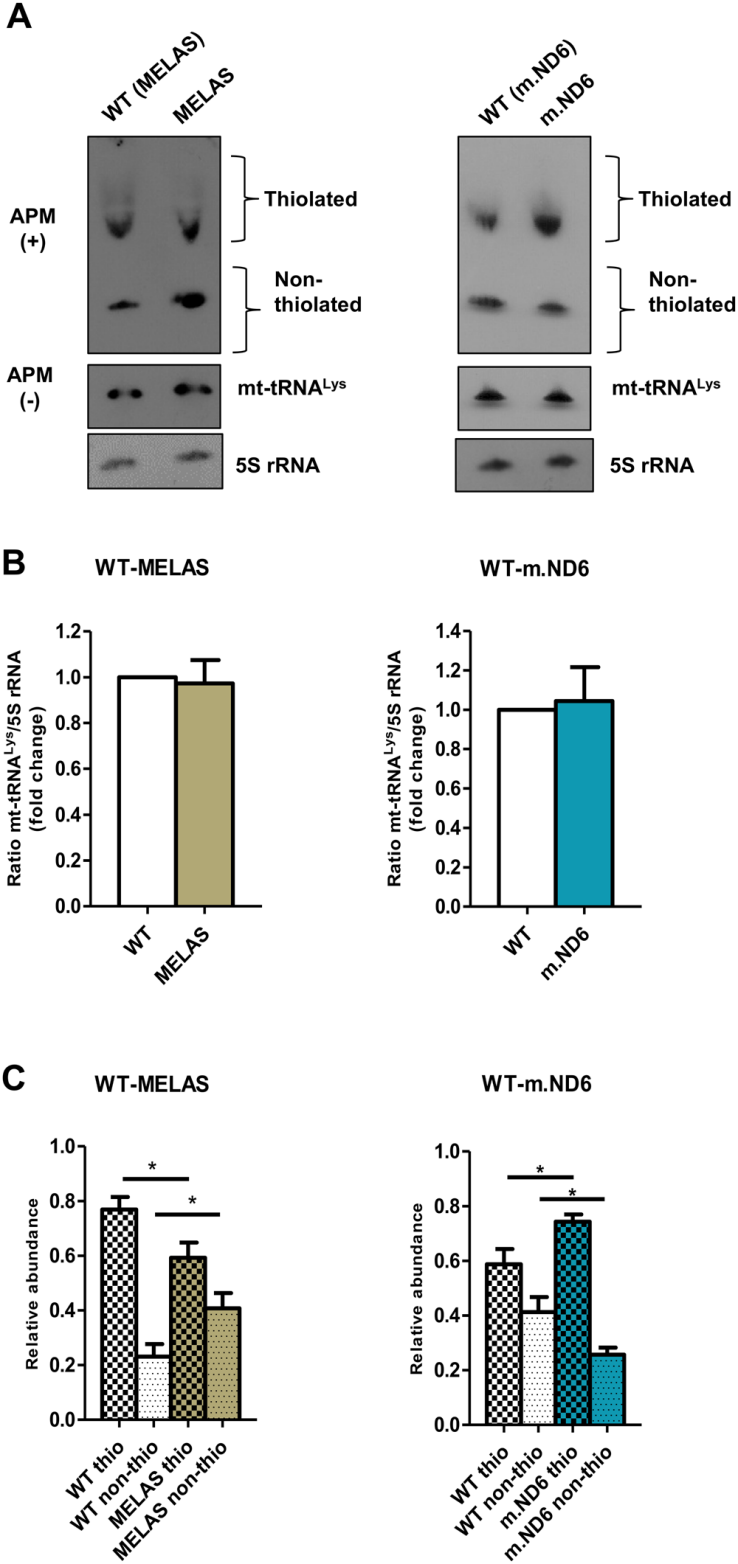
The 2-thiouridylation status of mt-tRNA^Lys^ depends on the TRMU expression level. (**A**) APM-Northern analysis of the 2-thiolation status of mt-tRNA^Lys^ obtained from mutant and WT cybrid cells. The same amount of total RNA (7.5 µg) was run in a denaturing polyacrylamide-urea gel in the presence (+) or absence (−) of APM. The thiolated tRNAs were detected as retarded bands in the presence of APM. The APM (−) membrane was also probed with 5S rRNA as a loading control. (**B**) Relative steady-state levels of mt-tRNA^Lys^. The signal corresponding to the amount of mt-tRNA^Lys^ in the APM (−) membrane was normalized to the signal corresponding to the amount of 5S rRNA and represented as fold change relative to WT. Full-length blots and lower-exposure blots for the APM (+) membranes are included in supplementary information. (**C**) Percentage of thiolated and nonthiolated mt-tRNA^Lys^ species compared with the whole amount of this mt-tRNA. The quantification of each fraction (thiolated or nonthiolated) is expressed as a percentage of its signal from the total signal (thiolated + non-thiolated signals). All data represent the mean ± SD of at least three different experiments. Differences from WT values were found to be statistically significant at *p < 0.05.

To explore the direct effects of the deregulation of the enzymes, we analyzed the thiolation status of mt-tRNA^Lys^ in MELAS and m.ND6 cybrid lines, in which protein TRMU was found to be down- and up-regulated, respectively (Fig. 2E). To this end, we used APM-Northern blotting, which relies on the retardation of thiolated tRNA molecules because of the affinity of the thio group for the p-(N-acrylamino)-phenylmercuric chloride (APM) compound in the gel^35^. As shown in Fig. 3, the non-thiolated mt-tRNA^Lys^ fraction was increased in MELAS (where TRMU is down-expressed) and reduced in m.ND6 (where TRMU is up-expressed). Our data also indicate that mt-tRNA^Lys^ is partially 2-thiolated even in WT cells, a condition that has been observed in other scenarios^36^, and support the idea that the non-thiolated/thiolated ratio of mt-tRNA^Lys^ molecules depends on the expression level of TRMU in each cybrid line. Although we observed a direct correlation between the levels of TRMU and 2-thiolation, there is no perfect match between fold-changes for both parameters. This could be due to the semi-quantitative nature of the APM-Northern and Western blotting analysis, to a higher stability of thiolated mt-tRNA molecules, which could affect the non-thiolated/thiolated ratio, and/or to features of the TRMU protein that remain to be determined.

### The cybrid lines exhibit a differential expression of ROS-sensitive miRNAs that target the U34 modification enzymes

We previously demonstrated that the ROS-sensitive microRNA-9/9* down-regulates the expression of GTPBP3, MTO1 and TRMU in MELAS cells^4^. To select additional, putative ROS-sensitive microRNAs that may regulate the expression of these proteins in the different cybrid models, we created a list of microRNAs predicted to bind to the 3′UTR region of the GTPBP3, MTO1 and TRMU mRNAs according to three databases (miRanda, miRWalk and Targetscan). From this list, we only kept microRNAs associated with an oxidative stress scenario^4^, which then were classified by the number of targeted enzymes (in order to identify putative miRNAs that can control simultaneously several U34 modification enzymes) and, subsequently, by the number of databases showing the microRNA-target interaction (Table S1). We selected miR-9/9*, miR-335/335* and miR-338-3p for experimental validation as they presented the highest total scores.

**Figure 4:**
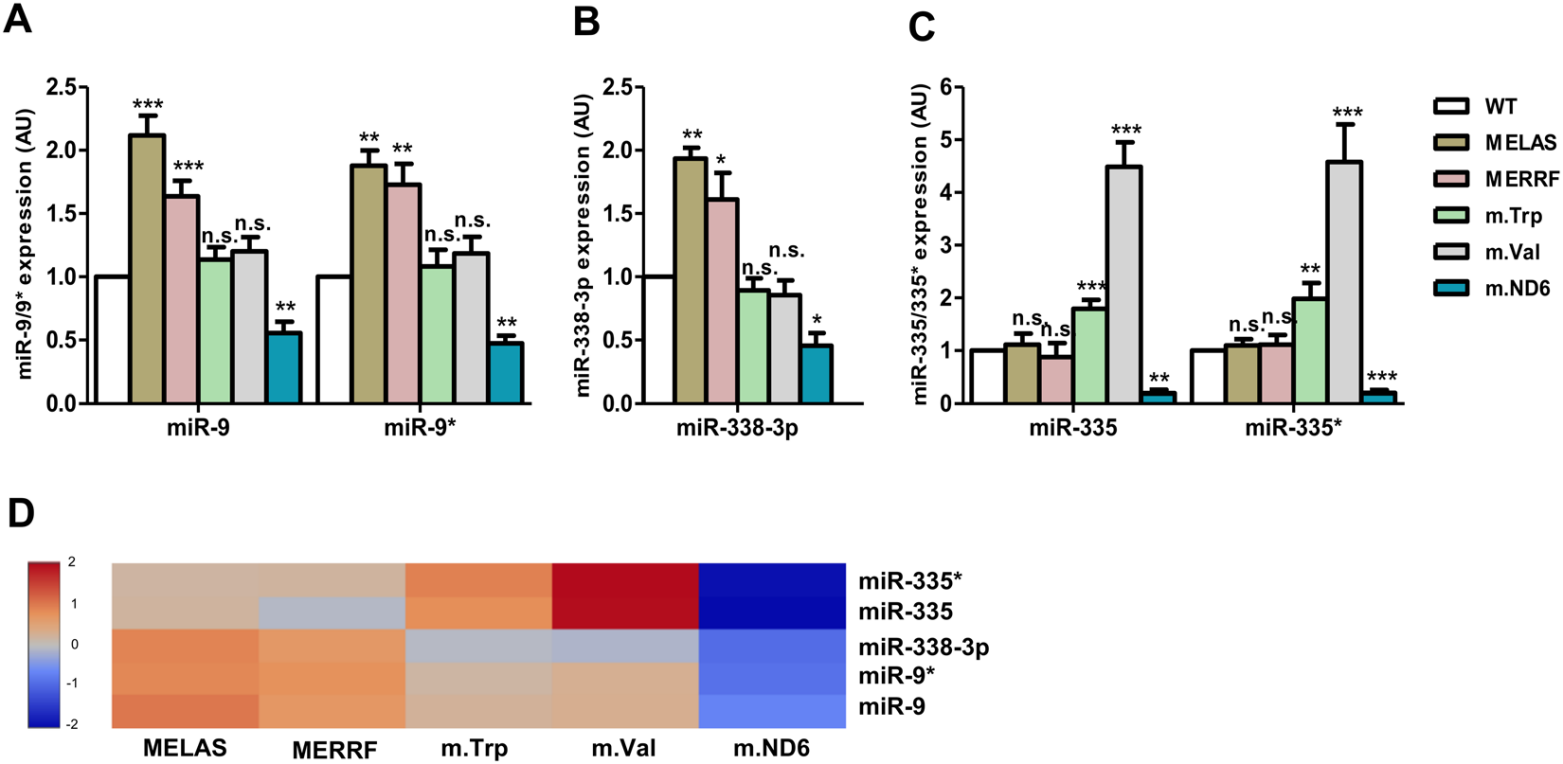
Mutation-dependent expression of miRNAs that are predicted to target the U34 modification enzymes. (**A–D**) qRT-PCR analysis of the miR-9/9* (**A**), miR-338-3p (**B**), and miR-335/335* (**C**) expression in mutant and WT cybrid cells. Results from this analysis are also shown as a heatmap (**D**). The color and the corresponding value in log_2_ scale are depicted on the left. All data are the mean ± SEM of at least three different experiments. Differences from WT values were found to be statistically significant at *p < 0.05, **p < 0.01 and ***p < 0.001. AU: arbitrary units.

Expression of miR-9/9* was found to be induced in MELAS and MERRF cells (Fig. 4A and D), as expected^4^. However, no changes in the miRNA-9/9* levels were observed in m.Trp and m.Val cells, whereas a noticeable decrease in the expression of this miRNA was found in m.ND6 cells (Fig. 4A and D). miR-338-3p followed the expression pattern of miR-9/9* (compare Fig. 4A and B, or see Fig. 4D), although its absolute abundance was lower in all cybrid lines, as inferred from qRT-PCR data (see Materials and Methods).

Since the expression of miR-9/9* and miR-338-3p is not affected in m.Trp and m.Val, other miRNA(s) could be responsible for the decrease of the GTPBP3 and MTO1 levels observed in these cells (Fig. 2E). We thought that miR-335/335* could be a good candidate given that the 3′ UTR region of *GTPBP3* and *MTO1*, but not that of *TRMU* (whose expression is increased in m.Trp and m.Val), have predicted binding sites for this miRNA (Table S1). Indeed, we found that the expression of miR-335/335* was induced in m.Trp and m.Val, remained unaltered in MELAS and MERRF, and was drastically reduced in m.ND6 (Fig. 4C and D).

**Figure 5:**
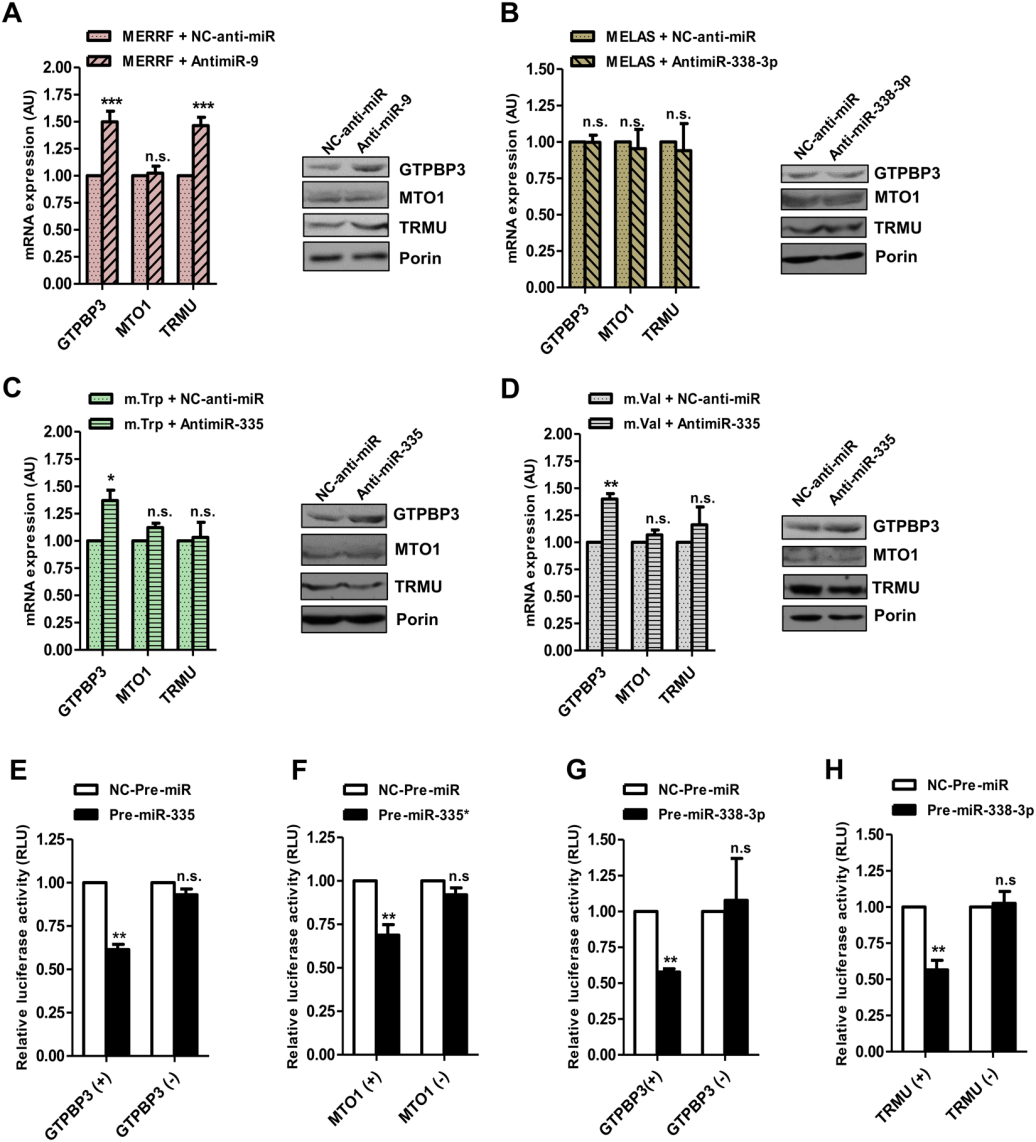
miR-9/9*, miR-338-3p, and miR-335/335* are regulators of the U34 modification enzymes. **(A–D)** qRT-PCR (left) and Western blot (right) analysis of *GTPBP3, MTO1* and *TRMU* expression in anti-miR-9-transfected MERRF cells (**A**), anti-miR-338-3p-transfected MELAS cells (**B**), anti-miR-335-transfected m.Trp cells (**C**) and anti-miR-335-transfected m.Val cells (**D**), and in the respective negative control (NC)-transfected cells (**A–D**). Full-length western blots are included in supplementary information. **(E)** Effects of miR-335 transfection on the activity of luciferase reporter constructs containing the GTPBP3-3′-UTR in the direct (+) or reverse (−) direction. (**F**) Effects of miR-335* transfection on the activity of luciferase reporter constructs containing the MTO1-3′-UTR in the direct (+) or reverse (−) direction. (**G** and **H**) Effects of miR-338-3p transfection on the activity of luciferase reporter constructs containing the GTPBP3-3′-UTR (**G**) and TRMU-3′-UTR (**H**) in the direct (+) or reverse (−) direction. All data are the mean ± SEM of at least three different experiments. Differences from NC values were found to be statistically significant at *p < 0.05, **p < 0.01 and ***p < 0.001. RLU: Relative light units.

Next we evaluated the regulatory role of miR-9/9*, -338-3p and -335/335* in the expression of their putative targets. First, we explored whether transfection of each cybrid model with a miRNA antagonist (anti-miR) led to the recovery of the targeted genes. Previous results indicated that transfection of the MELAS cybrid line with anti-miR-9 led to a noticeable increase in the mRNA and protein levels of GTPBP3 and TRMU^4^. Here, we observed that transfection of MERRF cells with anti-miR-9 also caused a clear accumulation of the *GTPBP3* and *TRMU* mRNAs (Fig. 5A). No changes in the expression of *MTO1* were found in the MERRF transfected cells (Fig. 5A), as expected given that *MTO1* is a direct target of miR-9* but not of miR-9^4^. In addition, we transfected MELAS cells with anti-miR-338-3p and no changes were observed in the mRNA expression of the putative miR-338-3p targets, *GTPBP3* and *TRMU* (Fig. 5B), which could be due to the low expression level of miR-338-3p in the cybrids. Finally, we found a recovery of the *GTPBP3* expression after transfection of m.Trp and m.Val with anti-miR-335 (Fig. 5C and D), which is in agreement with the prediction that this gene, but not *MTO1* and *TRMU*, is a target of miR-335 (Table S1).

Second, we explored the capability of miRNAs to bind to their predicted targets. As we previously demonstrated that *GTPBP3* and *TRMU* are directly targeted by miR-9, whereas MTO1 is targeted by miR-9*^4^, we focused here on testing the binding of miR-335/335* and miR-338-3p to their targets by means of luciferase reporter assays. To this end, we cloned the 3′UTR of the respective mRNAs in direct (+) or reverse (−) direction downstream of the luciferase reporter gene. Then, we co-transfected these plasmids into Hela cells together with the respective microRNA mimic (Pre-miR) or its negative control (NC-Pre-miR). The co-transfection of the GTPBP3 and MTO1 reporters together with miR-335 or miR-335* mimic, respectively, caused a decrease of over 30% in the luciferase activity as compared with the negative control-transfected cells (Fig. 5E and F). Moreover, the co-transfection of the GTPBP3 and TRMU reporters together with miR-338-3p mimic resulted in a decrease of about 50% in the luciferase activity (Fig. 5G and H). These effects were lost when assays were performed with reporters carrying the 3′UTR of each gene in the reverse direction (Fig. 5E–H). Altogether these data indicate that the selected miRNAs indeed bind to their predicted targets.

Briefly, our results demonstrate that miR-9/9* is a direct regulator of the *GTPBP3*, *MTO1* and *TRMU* expression in MERRF cells, whereas miR-335/335* controls the expression of *GTPBP3* and *MTO1* in m.Trp and m.Val cells. Despite miR-338-3p targets *GTPBP3* and *TRMU*, this miRNA does not appear to play an important role in the regulation of these genes in the MELAS and MERRF cybrids, likely because of its low expression level in these cells.

### miR-9 and miR-335 contribute to worsen the phenotype of specific cybrid models

**Figure 6:**
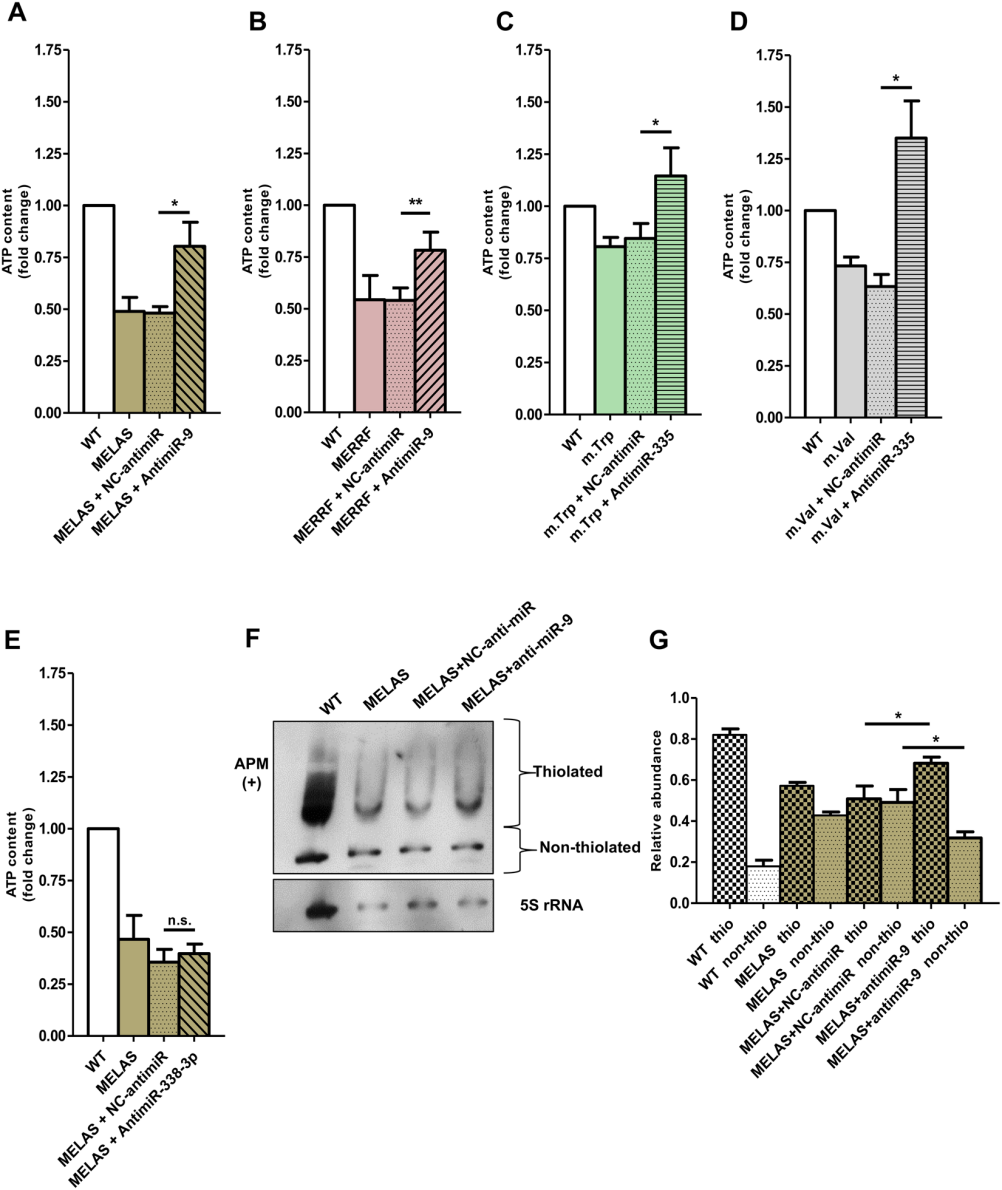
The induction of miR-9/9*, miR-338-3p and miR-335/335* worsens the energetic state of the cybrid lines. (**A–E**) Cellular ATP determination in anti-miR-9 transfected MELAS cells (**A**), anti-miR-9-transfected MERRF cells (**B**), anti-miR-335-transfected m.Trp cells (**C**), anti-miR-335-transfected m.Val cells (**D**), anti-miR-338-3p-transfected MELAS cells (E) and in the respective negative control (NC)-anti-miR transfected cells (**A–E**). **(F)** APM-Northern analysis of the 2-thiolation status of mt-tRNA^Lys^ obtained from MELAS cells transfected with either anti-miR-9 or NC-anti-miR. The thiolated tRNAs were detected as retarded bands in the presence of APM. The membrane was also probed with 5S rRNA as a loading control. Full-length blots and a lower-exposure blot of mt-tRNA^Lys^ are included in supplementary information. **(G)** Percentage of thiolated and nonthiolated mt-tRNA^Lys^ species compared with the whole amount of this mt-tRNA. The quantification of each fraction (thiolated or nonthiolated) is expressed as a percentage of its signal from the total signal (thiolated + non-thiolated signals). All data are the mean ± SEM of at least three different experiments. Differences from negative control (NC) values were found to be statistically significant at *p < 0.05 and **p < 0.01.

Downregulation of GTPBP3, or MTO1 or TRMU by siRNAs has been previously shown to affect mitochondrial protein synthesis, cellular ATP content, mitochondrial membrane potential, and oxygen consumption^37–39^. To evaluate the functional effect of down-regulation of the U34 modification enzymes mediated by miRNAs, we analyzed the effects produced by either the transfection of cybrid cells with anti-miRs or the transfection of wild-type cells with pre-miRs. As shown in Fig. 6, the ATP levels of MELAS and MERRF cells or m.Trp and m.Val cells increased significantly after transfection with anti-miR-9 (Fig. 6A and B) or anti-miR-335 (Fig. 6C and D), respectively. No increase of the ATP levels was observed after transfection of MELAS cells with anti-miR-338-3p (Fig. 6E). Altogether these data indicate that up-regulation of miR-9/9* in MELAS and MERRF cells, and of miR-335/335* in m.Val and m.Trp cells contributes to their energetic deficit, and that miRNA-338-3p plays an irrelevant role in the phenotype of MELAS and MERRF cybrids.

The increase in the ATP levels observed in MELAS cells after transfection with anti-miR-9 (Fig. 6A) correlates with an increase of both the TRMU expression^4^ and the 2-thiolation levels of mt-tRNA^Lys^ (Fig. 6F and G). Moreover, the decrease in the ATP levels observed in wild-type cells after transfection with pre-miR-9 (Fig. S2A) correlates with a decrease of the *TRMU* expression and 2-thiolation levels of mt-tRNA^Lys^ (Fig. S2B–D). Interestingly, transfection of the wild-type cells with pre-miR-9 or TRMU-siRNAs produces similar effects: a decrease in the *TRMU* expression, a decrease in the thiolation levels of mt-tRNA^Lys^, and a decrease in the cellular ATP levels (Figs S2 and S3; see also ref. 4). Altogether these data suggest that the functional effect of miR-9 occurs, at least partially, through the mt-tRNA modification function of TRMU.

### Critical thresholds of ROS and Ca^2+^ determine the expression levels of miR-9 and miR-335*

**Figure 7:**
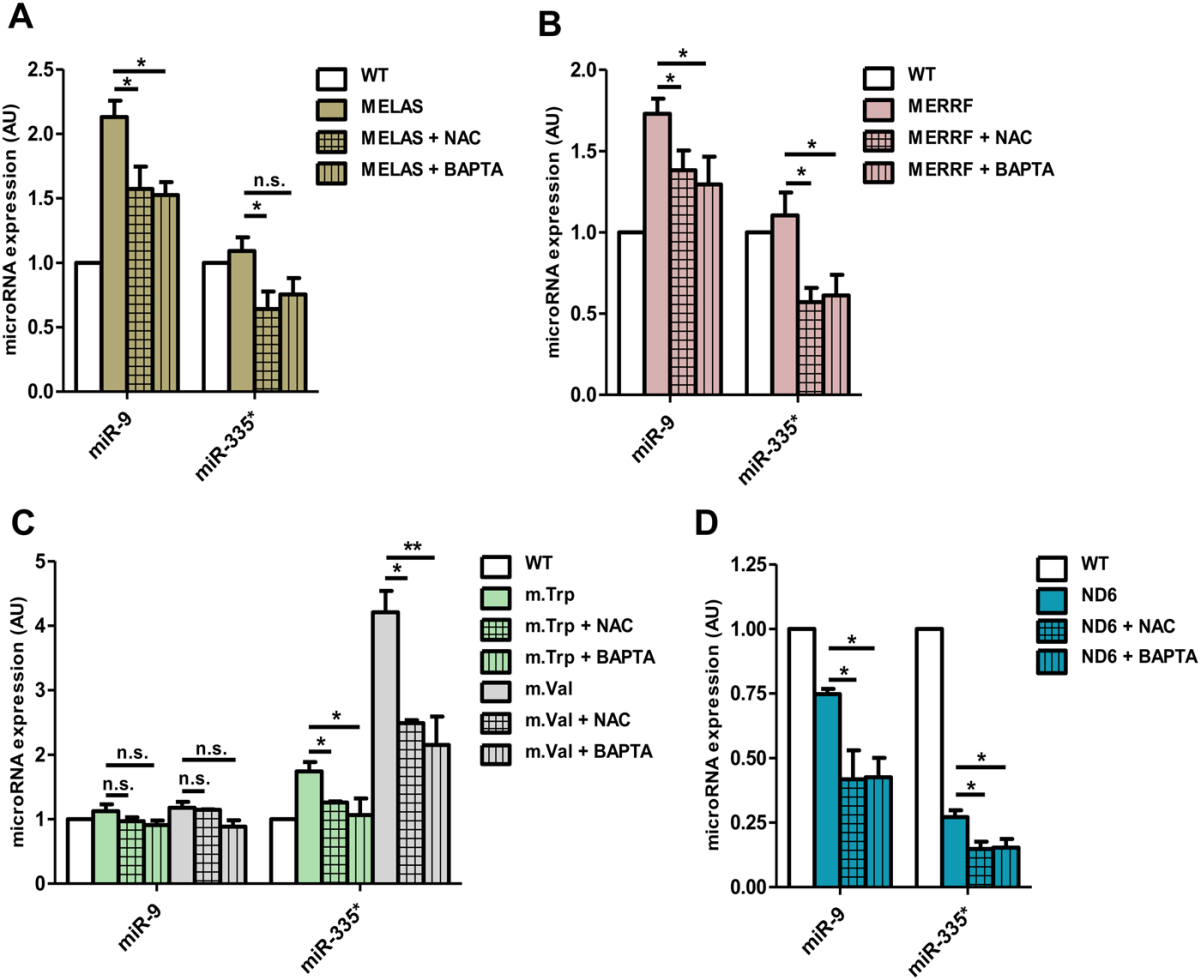
Elevated levels of ROS and intracellular Ca^2+^ stimulate the expression of miR-9/9* and miR-335/335* in mutant cybrid cells. (**A–D**) qRT-PCR analysis of miR-9 and miR-335* expression in MELAS (**A**), MERRF (**B**), m.Trp, m.Val (**C**) and m.ND6 (**D**) cybrid cells treated with either 1 mM NAC (antioxidant) for 48 h or 10 μM BAPTA (Ca^2+^ chelator) for 2 h. All data are the mean ± SEM of at least three different experiments. Differences from WT values were found to be statistically significant at *p < 0.05 and **p < 0.01.

It is striking that the expression of GTPBP3 and MTO1 is controlled by miR-9/9* in MELAS and MERRF cybrids, and by miR-335/335* in m.Trp and m.Val cells. We asked whether the different expression pattern of miRNAs among the cybrid lines could depend on threshold levels of retrograde signals like ROS and Ca^2+^. To address this question, we first analysed the effects of the antioxidant N-acetyl-cysteine (NAC) and the intracellular Ca^2+^ chelator 1,2-Bis(2-aminophenoxy)ethane-N,N,N′,N′-tetraacetic acid tetrakis(acetoxymethyl ester) (BAPTA) on the expression of miR-9 and miR-335 in the cybrid lines. We found that the reduction of ROS and Ca^2+^ levels by NAC and BAPTA treatments (Fig. S4A and B, respectively) caused a decrease in the induced levels of miR-9 in MELAS and MERRF cybrids (Fig. 7A and B) and of miR-335* in m.Trp and m.Val cells (Fig. 7C). In addition, we observed that, despite the decreased expression of miR-9 in ND6 cells (Fig. 7D) and the uninduced or very low levels of miR-335* in MELAS, MERRF and ND6 (Fig. 7A,B and D), the expression of both miRNAs could be further reduced after treatment of the cells with the antioxidant (NAC) or Ca^2+^ chelating (BAPTA) agent. A reduction in the miR-9 and miR-335 levels was also observed in WT cybrids treated with these agents (Fig. S6). These data support the idea that certain minimum threshold levels of ROS and Ca^2+^ are required for wild-type expression of miR-9 and miR-335*.

**Figure 8:**
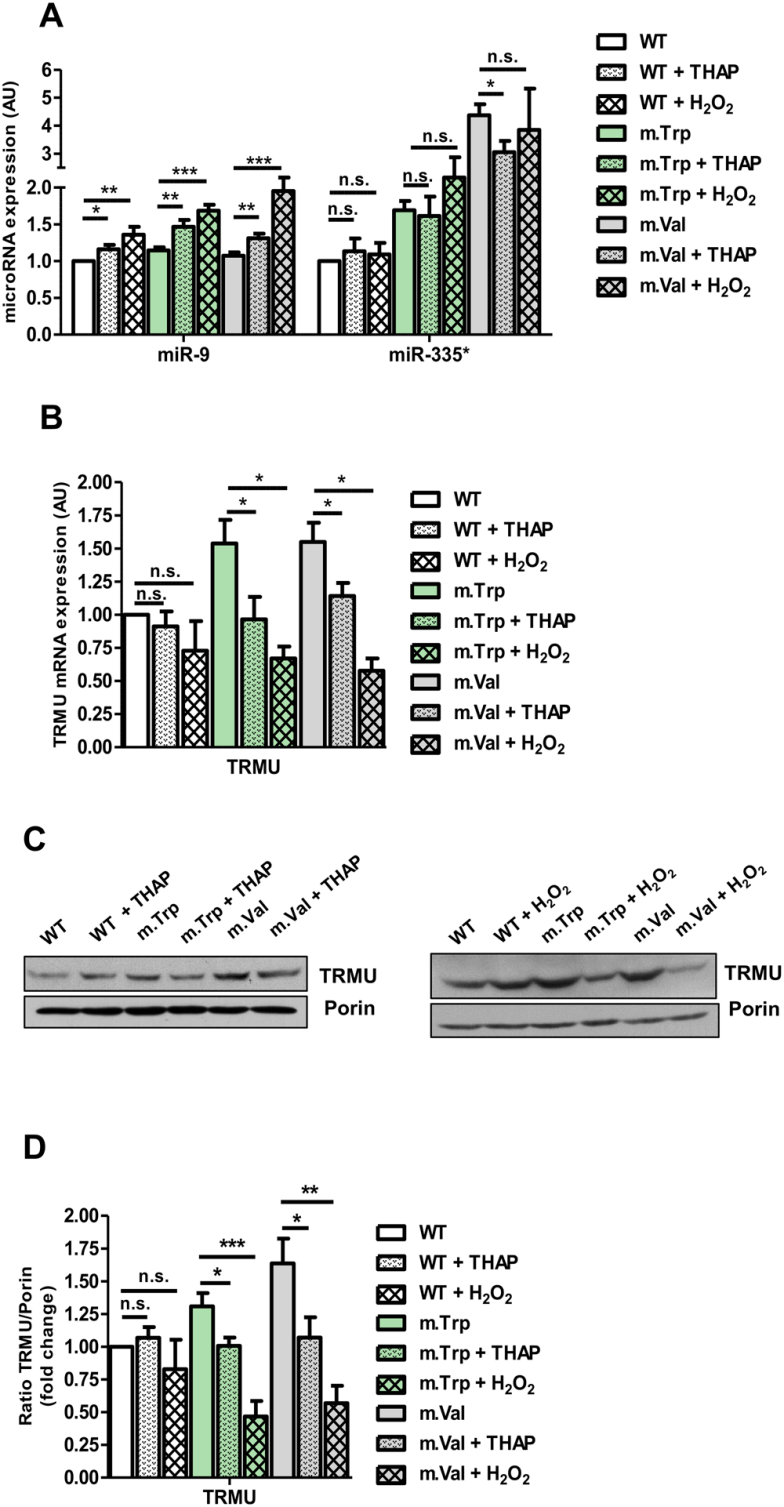
Treatment of m.Trp and m.Val cybrids with Thapsigargin or H_2_O_2_ triggers the expression of miR-9 and reduces the TRMU levels. (**A**) qRT-PCR analysis of miR-9 and miR-335 expression in m.Trp, m.Val and WT cybrid cells treated with either 400 nM Thapsigargin (THAP) for 24 h or 5 mM H_2_O_2_ for 6 h. (**B**) qRT-PCR analysis of TRMU mRNA expression in m.Trp, m.Val and WT cybrid cells treated with either 400 nM Thapsigargin (THAP) for 24 h or 5 mM H_2_O_2_ for 6 h. (**C**) Representative western blot of the expression of TRMU in m.Trp, m.Val and WT cybrid cells treated with either 400 nM Thapsigargin (THAP) for 24 h (left) or 5 mM H_2_O_2_ for 6 h (right). The membrane was also probed with porin as a loading control. Full-length western blots are included in supplementary information. (**D**) Densitometric analysis of TRMU normalized to porin and represented as fold change relative to WT. All data are the mean ± SEM of at least three different experiments. Differences from WT values were found to be statistically significant at *p < 0.05, **p < 0.01 and ***p < 0.001.

Next, considering that the ROS and Ca^2+^ levels reached in MELAS and MERRF cybrids were higher than in m.Trp and m.Val cells (Fig. 1F and G), we evaluated the effect of increasing the levels of ROS or Ca^2+^ in the m.Trp and m.Val cybrids by treatment with H_2_O_2_^40, 41^ or Thapsigargin^42^. Notably, the first one increased the levels of both ROS and Ca^2+^ (Figs S5A and S5B), whereas Thapsigargin only had an effect on the Ca^2+^ levels (Figs S5C and S5D). Interestingly, we found that miR-9 was induced in m.Trp and m.Val cells after both treatments, although the induction was higher in the H_2_O_2_-treated cells (Fig. 8A, left), and that the effect of Thapsigargin and H_2_O_2_ was quenched by pre-treatment of cells with BAPTA and NAC, respectively (Fig. S7), which indicates that the induction of miR-9 is mediated by the levels of Ca^2+^ and ROS. In contrast, the expression of miR-335* in the m.Trp and m.Val cells was not further increased after treatment with Thapsigargin or H_2_O_2_ (Fig. 8A, right). These data indicate that the increase of ROS and/or Ca^2+^ levels in m.Trp and m.Val cells promotes the induction of miR-9, while leaving roughly unaffected the high expression of miR-335*. It should be mentioned that treatment of MELAS cells with Thapsigargin or H_2_O_2_ did not affect significantly the expression of miR-9 and miR-335^*^ (Fig. S8), suggesting that the endogenous levels of ROS and Ca^2+^ in MELAS cells include the maximum threshold required for induction of miR-9.

The fact that miR-335* remains highly induced in Thapsigargin- or H_2_O_2_-treated m.Trp and m.Val cells (i.e., in the presence of increased levels of ROS and Ca^2+^) (Fig. 8A), while its expression is not induced in the MELAS and MERRF cells (Fig. 4C and D) suggests that miR-335* requires an additional, still unknown, signal to be induced, which is not present in MELAS and MERRF cells.

We have previously demonstrated that oxidative stress mediates the induction of miR-9/9* via an NFkB-dependent signaling pathway^4^. NFkB activation is initiated by the signal-induced degradation of IkB repressor proteins, which triggers the translocation of NFkB from the cytoplasm to the nucleus, thus activating the transcription of the target genes, like *miR-9*. We found that the IKBα expression is compromised not only in MELAS but also in MERRF cells, whereas it remains unaffected in m.Trp, m.Val, and m.ND6 (Fig. S9). These data suggest that induction of miR-335/335*, unlike that of miR-9/9*, is independent of NFkB. As expected, the treatment of m.Trp, and m.Val cybrids with H_2_O_2_ or Thapsigargin led to a decrease in the IKBα expression (Fig. S10), which correlates well with the induction of miR-9 observed in the treated cells (Fig. 8A).

In order to analyze the biological consequences of the induction of miR-9 in the H_2_O_2_- and Thapsigargin-treated m.Trp and m.Val cells, we assessed the expression of TRMU since this gene is a target of miR-9, but not of miR-335/335* (Table S1). The miR-9 induction in H_2_O_2_- or Thapsigargin-treated m.Trp and m.Val cells produced a concomitant decrease of TRMU expression, being more acute in H_2_O_2_-treated cells (Fig. 8B–D). These data are in line with the idea that threshold levels of ROS and Ca^2+^ induce miR-9/9* and modify the expression pattern of nucleus-encoded proteins, including the mt-tRNA modification enzymes.

## Discussion

Modifications at wobble uridines in tRNAs have been shown to regulate global protein expression by promoting selective translation of codon-biased mRNAs^8, 22, 43^, influencing nascent chain folding^44^, and contributing to signaling functions of tRNAs^45–47^. There is emerging evidence for reprogramming of U34 modifications in response to stress^4, 22, 48^. However, the regulatory mechanisms controlling the expression of tRNA modification enzymes and their role in the modification reprogramming are poorly known. In this study, we demonstrate that the expression of the mt-tRNA modifying enzymes GTPBP3, MTO1 and TRMU varies in response to different pathological mtDNA mutations, thus altering the modification status of mt-tRNAs. Importantly, we demonstrate that the expression of these enzymes is regulated by different miRNAs, whose activation depends on retrograde stress signals like ROS and Ca^2+^.

We used five different cybrid models of mtDNA mutations: four of them carry mutations in mt-tRNA genes, while the fifth contains a mutation affecting the complex I subunit ND6. In all mutant cybrid lines, including m.ND6, we observed a differential induction of mitoproteases involved in protein quality control and mitoribosome assembly, and an altered expression of GTPBP3, MTO1 and TRMU. Our data suggest that the up- or down-regulation of the U34 modification enzymes is part of a cellular response to cope with a stoichiometric imbalance between mtDNA- and nDNA-encoded OXPHOS subunits, which causes proteostasis stress.

Recent data indicate that the U34 of mt-tRNA^Lys^, mt-tRNA^Glu^ and mt-tRNA^Gln^ from mouse hepatocytes is not fully modified^36^. In agreement with this, our data show that mt-tRNA^Lys^ from wild-type cybrids is partially thiolated. Therefore, there is a chance that the non-modified/modified ratio of the mt-tRNA substrates for TRMU, GTPBP3 and MTO1 can be increased or decreased in response to stress, and that regulation of this ratio depends on the expression levels of the U34 modification enzymes. Both an increase and a decrease of mitochondrial translation may be involved in stress responses. Notably, it has been shown that the modifications introduced by the *E. coli* orthologs of TRMU (at position 2 of U34), and GTPBP3 and MTO1 (at position 5 of U34) modulate differently the relative efficiency of anticodons in reading cognate codons^49^. Moreover, the 2-thio group, but not the group introduced by the GTPBP3 and MTO1 orthologs, is important for aminoacylation^50^. These roles of the bacterial U34 modifications could be conserved by their counterparts in mt-tRNAs. Furthermore, we cannot rule out the possibility that U34 modifications may increase stability of mt-tRNAs. The different roles played by these modifications could explain the differential expression of the U34 modification enzymes in some cybrids. Thus, it is possible that the two types of U34 modifications (TRMU- and GTPBP3/MTO1-dependent) regulate mitochondrial translation according to the particular perturbation of proteostasis caused by each mtDNA mutation.

There are evidences, from a mouse model lacking a protein directly involved in mitochondrial translation, that the activation of adaptive responses to the impairment of mitochondrial proteostasis precedes respiratory chain deficiency during the development of mitochondrial diseases^51^. Our results clearly show that the five mutant cybrid lines differ in the expression of the mitoproteases LONP1, AFG3L2 and CLPP, which suggests that each mutation triggers an adaptive response to compensate the perturbation in the stoichiometric balance between components of OXPHOS complexes. Apparently, these adaptive responses are not able to restore the mitonuclear balance in the MELAS, MERRF, m.Trp and m.Val cybrids, since all of them exhibit a strong decrease in the steady-state levels of at least complex IV, and increased levels of ROS and Ca^2+^. These retrograde signals unleash a second wave of nuclear responses, including up- or down-regulation of miRNAs involved in the control of the U34 modification enzymes, thus altering the ratio of modified/unmodified mt-tRNA molecules. Variations in the modification state of the U34 in mt-tRNAs may affect both the global mitochondrial translation as well as putative non-conventional functions of mt-tRNAs (for instance, cell signaling). These successive waves of stress responses fail, however, to provide full protection from the OXPHOS dysfunction; rather, they appear to aggravate some phenotypic traits of the disease cell models since the transfection of these cybrids with appropriate anti-miRs improves the energetic state of the cells.

Interestingly, GTPBP3 and MTO1 are down-regulated in all cybrid lines carrying mutations in mt-tRNA genes (MELAS, MERRF, m.Trp, and m.Val cells), although down-regulation occurs via different miRNAs (miR9/9* in MERRF and MELAS; miR335/335* in m.Trp and m.Val). Our data indicate that both miR-9/9* and miR-335/335* require relatively high levels of ROS and Ca^2+^ to be induced, as the treatment of the MELAS, MERRF, m.Trp and m.Val with the antioxidant agent NAC or the Ca^2+^ chelator BAPTA reduced the expression of the up-regulated miRNAs. Our data also show that the NFkB-dependent induction of miR-9/9* requires threshold levels of ROS and/or Ca^2+^, given that the increase of these signals by treatment with H_2_O_2_ or Thapsigargin led to the induction of miR-9 in m.Trp and m.Val cells without severely affecting the high expression levels of miR-335. However, the induction of miR335/335* requires an additional factor(s) that remains to be identified, as the elevated levels of ROS and Ca^2+^ inherent to the MERRF and MELAS cybrids did not lead to the induction of this miRNA in both cybrid lines. The unaltered expression of the IkBα repressor found in the m.Trp and m. Val cybrids suggests that NFkB is not responsible for the induction of miR-335/335*. Putative regulators of this miRNA are the transcription factor CREB^52^ and the cAMP/PKA pathway^53^. Further studies are needed to explore these possibilities and clarify the miR-335/335* induction pathway.

TRMU is down-regulated in MELAS and MERRF cybrids, but up-regulated in m.Trp and m.Val cells, at both mRNA and protein levels. TRMU is a target of miR-9, but the levels of this miRNA in m.Trp and m.Val cells are similar to those found in the WT cybrid. Thus, the TRMU up-regulation in m.Trp and m.Val does not appear to depend on the miR-9 levels. It is worthy to note that TRMU is a target of other miRNAs (Table S1), which have not been included in this study due to the selection criteria. Therefore, it is possible that some of these miRNAs are down-regulated by the retrograde signaling pathway(s) activated in the mt.Trp and mt.Val cells, which would lead to the TRMU mRNA overexpression. Alternatively, the TRMU induction in the mt.Trp and mt.Val cells could be regulated at transcriptional levels. The biological meaning behind the induction of TRMU in a context (m.Val and m.Trp cells) where GTPBP3 and MTO1 are down-regulated remains unclear, but it could be related to new, still unknown functions of these enzymes and/or, as aforementioned, to the specific role of the modifications (s2 and τm5) introduced by the enzymes^10, 54, 55^.

The m.ND6 cells exhibited a behavior different from the rest of the mutant cybrids. No significant changes in the steady-state levels of the OXPHOS complexes were observed in the m.ND6 cells, although proteases LONP1, AFG3L2 and CLPP were found to be overexpressed. Moreover, there was a clear tendency to the accumulation of proteins TRMU, GTPBP3 and MTO1 in these cells, despite the mRNA levels were similar to those found in the WT cybrids. These data suggest that either the translation or the stability of the U34 modification enzymes is improved in the m.ND6 cells. Interestingly, the expression of miRNA-9/9*, miRNA-338-3p and, particularly, miRNA-335/335* was severely down-regulated in these cells, which could be part of a program to guarantee a high expression level of GTPBP3, MTO1 and TRMU in the genetic context of the m.ND6 cybrid line.

In brief, it appears that cells may function normally with a certain level of hypomodified mt-tRNAs, and that variations in this level represent a mechanism to regulate expression of mtDNA-encoded proteins as an adaptive response to the impairment of mitochondrial proteostasis. This work demonstrates the crucial role of miRNAs in the regulation of TRMU, GTPBP3 and MTO1, and, accordingly, in the modification status of the mt-tRNAs.

## Methods

### Materials

N-acetylcysteine, Thapsigargin and Hydrogen peroxide (H_2_O_2_) were purchased from Sigma. 1,2-Bis(2-aminophenoxy)ethane-N,N,N′,N′-tetraacetic acid tetrakis(acetoxymethyl) ester (BAPTA-AM) was purchased from Tocris. The APM ([p-(N-acrylamino)-phenyl]mercuric chloride) was synthesized and kindly provided by Prof. Stephane Vincent^56^. Oligonucleotides (Table S2) were purchased from Sigma and Qiagen.

### Cell culture

Transmitochondrial cytoplasmic hybrids (cybrids) were previously generated by fusion of platelets, derived from five patients carrying m.3243 A > G, m.8344 A > G, m.5514 A > G, m.1643A > G and m.14487 T > C mutations, respectively, with human osteosarcoma 143B cells lacking mtDNA (ρ0 cells). Briefly, platelet cells were isolated from a blood sample and fused to a large excess of mtDNA-less human osteosarcoma 143B (TK−) cells by using polyethylene glycol, as described^57^. Different cybrid clones were obtained by culturing the fusion mixture in selective Dulbecco’s modified Eagle’s medium (DMEM) (Biological Industries, Kibbutz Beit Haemek, Israel) containing glucose (4.5 g/l), pyruvate (0.11 g/l), 10% dialyzed fetal bovine serum (FBS) (Invitrogen, Carlsbad, CA, USA) and 100 μg/ml BrdU (5-bromo-2-deoxyuridine; without uridine) to prevent growth of TK + donor cells. Different clones were selected, DNA extracted from each clone and analyzed by polymerase chain reaction-restriction fragment length polymorphism (PCR-RFLP) to find cells containing 100% mutant mtDNA or the wild type counterpart (WT cybrids). The presence or absence of the mutation in the cells was periodically confirmed using a PCR-RFLP assay^24, 25, 58^. All cybrids were cultured in high glucose Dulbecco’s modified Eagle medium (Gibco) containing 10% fetal bovine serum, 1 mM sodium pyruvate, 100 U/ml penicillin, 100 μg/ml streptomycin, 2 mM glutamine and 1 mM non-essential amino acids. Human HeLa cells were grown in full medium: Minimum Essential Medium (MEM) (Sigma) supplemented with 10% heat-inactivated fetal bovine serum, 100 U/ml penicillin and 100 μg/ml streptomycin.

### Anti-miR and pre-miR transfection

Cells were seeded at 250,000 cells/well in 6 well-plate or at 1,500,000 cells/100mm dish. After 24 h, cells were transfected with one of the RNA oligonucleotides (Anti-miR-9, anti-miR-335, anti-miR-338-3p, Negative Control (NC)-anti-miR, pre-miR-338-3p, pre-miR-335, pre-miR-335* or Negative Control (NC)-pre-miR; Applied Biosystems) at the 50 nM final concentration, using Lipofectamine 2000 reagent (Invitrogen) and Opti-MEM medium according to the manufacturer’s instructions. After 6 h of transfection, the medium was changed to fresh growth medium. Cells were collected after 48 h of transfection.

### APM-Northern blotting analysis

The procedure was performed as previously described to assess the thiolation status of mitochondrial tRNAs^4^. Total RNA (7.5 μg) was run on a 15% polyacrylamide gel containing 7 M urea and 10 μg/ml APM and then transferred to positively charged nylon membranes (Roche). Pre-hybridization and hybridization steps were performed with Dig Easy Hyb (Roche) according to the manufacturer’s instructions. mt-tRNA^Lys^ was detected with a specific DIG-labeled synthetic oligodeoxynucleotide (Table S2). Quantification of the non-radioactive signals was performed with ImageQuant TL v8.1 (GE Healthcare Life Sciences). The fraction of thiolated and non-thiolated tRNAs was calculated as described^15^. Briefly, the quantification of each fraction was expressed as a percentage of the thiolated or the non-thiolated signal from the thiolated + non-thiolated signals (as detected on the (−) APM gel).

### Luciferase reporter assays

The luciferase reporter plasmids, containing the 3′UTRs of GTPBP3, MTO1 and TRMU genes, were obtained as described^4^. HeLa cells were seeded at 50,000 cells/well, and the following day, 500 ng of a Luciferase reporter plasmid and, as an internal control, 25 ng of Renilla Luciferase control vector (Promega) were co-transfected together with one of the RNA oligonucleotides at the 50 nM final concentration, using Lipofectamine 2000 reagent (Invitrogen) and Opti-MEM medium according to the manufacturer’s instructions. After 48 h, cells were lysed and Firely and Renilla luciferase activities from the cell extracts were measured with the Dual-luciferase Reporter Assay System (Promega) following the manufacturer’s procedure.

### RNA isolation and qRT-PCR

Total RNA was isolated using TRIzol reagent (Invitrogen). To quantify mRNA levels, one-step qRT-PCRs were performed in an Applied Biosystems Step-One Real-Time PCR System. 50-300 ng of total RNA were reverse-transcribed and amplified by qPCR in 20 μl of total volume reaction containing specific primers (Sigma), Power SYBR Green PCR Master Mix, MultiScribe Reverse Transcriptase, and RNase Inhibitor (all from Applied Biosystems), according to the manufacturer’s instructions. The efficiency values obtained for qPCR amplifications were very near to 2. Relative quantitation of mRNA levels was calculated using the comparative Ct method. ACTB gene was used as endogenous control. A list of the primers used in this work is provided in Table S2. The absolute abundances of GTPBP3, MTO1 and TRMU mRNAs were estimated to be in a range of 1 to 15 copies/cell in mutant cybrid cells by using equivalent calculations as described^4^. For miRNA quantification, 10 ng of total RNA were reverse-transcribed in 15 μl total reaction using the MultiScribe reverse transcriptase and specific stem-loop RT primers (Applied Biosystems). Then, 1.33 μL of cDNA was subjected to a TaqMan miRNA assay (Applied Biosystems), in a total reaction volume of 20 μL using specific primers and probes for human miR-9, miR-9*, miR-338-3p, miR-335, miR-335* and U6 snRNA, according to the manufacturer’s protocol. Expression values were calculated using the comparative CT method. U6 snRNA was used as an endogenous control. The absolute abundances of either miR-9, miR-9*, and miR-338-3p in MELAS and MERRF cybrid cells or miR-335 and miR-335* in m.Trp and m.Val cybrid cells were also estimated as described^4^. We estimated the abundance of miR-9 and miR-9* to be at about 2200 and 250 copies/cell, respectively. miR-338-3p was found at approximately 15 and 35 copies/cell in MELAS and MERRF cybrid cells, respectively, whereas miR-335 and miR-335* were at 280 and 260 copies/cell in m.Trp cybrid cells and at 810 and 700 copies/cells in m.Val cybrid cells. Only the amounts of miR-9, miR-9*, miR-335 and miR-335* in the cell exceed the necessary threshold level of miRNA expression (100 copies/cell) proposed for significant target suppression^59^.

### Measurement of mitochondrial DNA

mtDNA quantification was performed by qPCR as previously described^60^.

### Measurement of intracellular ATP

The amount of ATP was measured with the ATP Bioluminescence Kit Assay Kit HSII (Roche), following the manufacturer’s instructions. Measurements were performed after the treatment or 48 h after transfection. Briefly, cells were detached with trypsin-EDTA, resuspended with fresh medium, centrifuged at 100 g and resuspended again with kit dilution buffer at 10^6^ cells/mL. Then, the same volume of kit cell lysis reagent was added to the cell suspension and the mixture incubated for 5 min at room temperature. After transferring the appropriate volume of sample into a microwell plate well, the kit luciferase reagent was added. Luminescence was determined using the Spectra Max M5 (Molecular Devices).

### Flow cytometry studies

After the indicated treatments, cells were detached at 37 °C with trypsin-EDTA and resuspended in fresh growth media. For ROS analysis, cells were incubated with 5 μM MitoSOX Red for 30 min at 37 °C, washed twice with phosphate buffered saline (PBS), and the red fluorescence emitted by the dye (540–625 nm band-pass filter) was measured^39^. For intracellular calcium determination, cells were incubated with 5 μM Fluo-3-AM for 30 min at 37 °C, washed twice with phosphate buffered saline (PBS), and the emitted fluorescence (488 ± 20-nm band-pass filter) was recorded^61^. For all the measurements, 10,000 cells were analysed and collected using a Cytomics FC 500 flow cytometer (Beckman Coulter).

### Blue-Native PAGE

BN-PAGE was performed similarly as described^62^. In brief, mitoplasts were prepared by treatment with 1.2 mg digitonin per mg of protein, and were then solubilized with 1% lauryl maltoside, which is a mild non-ionic detergent that allows for separation of individual complexes (complexes I-V) rather than retaining the supercomplexes. Samples containing 10–20 µg of protein were separated on a 3–12% Bis-Tris Novex NativePAGE gel (Life Technologies). The relative level of the assembled respiratory complexes I-IV was assessed by Western blot with commercially antibodies: mouse monoclonal anti-NDUFB8 antibody (459210, Invitrogen), mouse monoclonal anti-SDHA antibody (ab14715, Abcam), mouse monoclonal anti-Complex III subunit Core 1 antibody (459140, Invitrogen) and rabbit polyclonal anti-COX IV antibody (459600, Invitrogen). Complex V was detected with a rabbit polyclonal antibody^63^.

### Western blot

Cell extracts were prepared in RIPA buffer (150 mM NaCl, 1% Nonidet P40, 0.5% sodium deoxycholate, 0.1% SDS and 50 mM Tris-HCl pH 8.0), containing 0.1 mM leupeptin and 1 mM phenylmethanesulphonyl fluoride. When phosphoproteins were assessed, cell extracts were prepared in lysis buffer (10 mM NaCl, 1% Nonidet P40, 15% Glycerol, and 50 mM Tris-HCl pH 7.4), containing 1 mM phenylmethanesulphonyl fluoride, 0.1 mM leupeptin, 2 mM sodium orthovanadate, 100 mM sodium fluoride and 20 mM tetrasodium pyrophosphate. Proteins (100 μg) from the various lysates were separated by SDS/PAGE (10% acrylamide) and transferred to PVDF membranes (GE Healthcare, Amersham Biosciences). For immunodetection, anti-GTPBP3 antibody was purified from GTPBP3-His-inoculated rabbit serum^39^. The others were commercial: rabbit polyclonal anti-MTO1 antibody (15650-1-AP, Protein tech), rabbit polyclonal anti-TRMU (sc-86923, Santa Cruz Biotechnology), rabbit polyclonal anti-porin antibody (ab15895, Abcam), rabbit polyclonal anti-LONP1 (NBP1-81734, Novus Biologicals), mouse polyclonal anti-AFG3L2 (ab68023, Abcam), rabbit monoclonal anti-CLPP (ab124822, Abcam), rabbit polyclonal anti-IKBα (sc-847, Santa Cruz Biotechnology). The anti-rabbit (A6154) and anti-mouse (A4416) IgG-horseradish peroxidase-conjugated secondary antibodies were obtained from Sigma. Protein bands were quantified by densitometric analysis with an Image Quant ECL (GE Healthcare).

### Statistical analysis

Statistical analysis was performed using Student’s t test. The statistically significant differences between the means were indicated by asterisks (*p < 0.05, **p < 0.01 or ***p < 0.001), and non-significant differences by n.s.

## Electronic supplementary material

Supplementary Info
