# Supplementary material for: microRNA-mediated differential expression of TRMU, GTPBP3 and MTO1 in cell models of mitochondrial-DNA diseases
Source: Sci Rep. 2017 Jul 24;7:6209. doi: 10.1038/s41598-017-06553-w (PMC5524753; doi:10.1038/s41598-017-06553-w)
Supplement: Supplementary file 1 — Supplementary Info [file 41598_2017_6553_MOESM1_ESM.pdf]

microRNA-mediated differential expression of TRMU, GTPBP3 and MTO1 in cell models of mitochondrial-DNA diseases

Salvador Meseguer, Olga Boix, Carmen Navarro-González, Magda Villarroya, Rachid Boutoual, Sonia Emperador, Elena García-Arumí, Julio Montoya, and M.-Eugenia Armengod

Supplementary Information

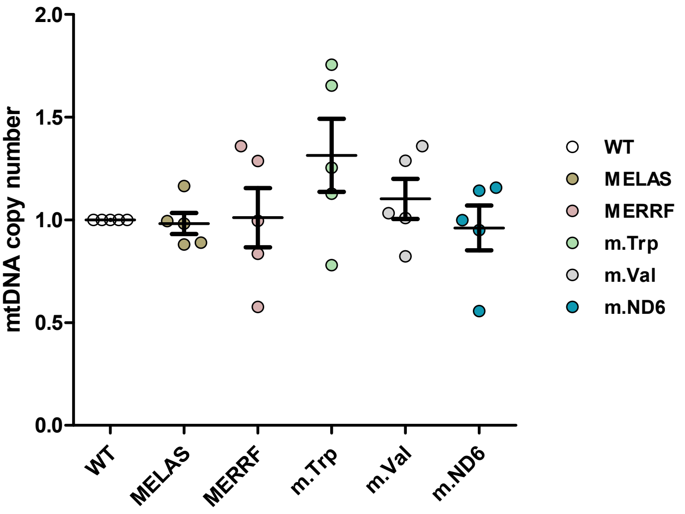

Figure S1. mtDNA copy number. Relative quantification of the mitochondrial-encoded *COXII* gene to the nuclear-encoded *SDH* gene in mutant and WT cybrid cells. All data are the mean ± SEM of at least three different experiments.

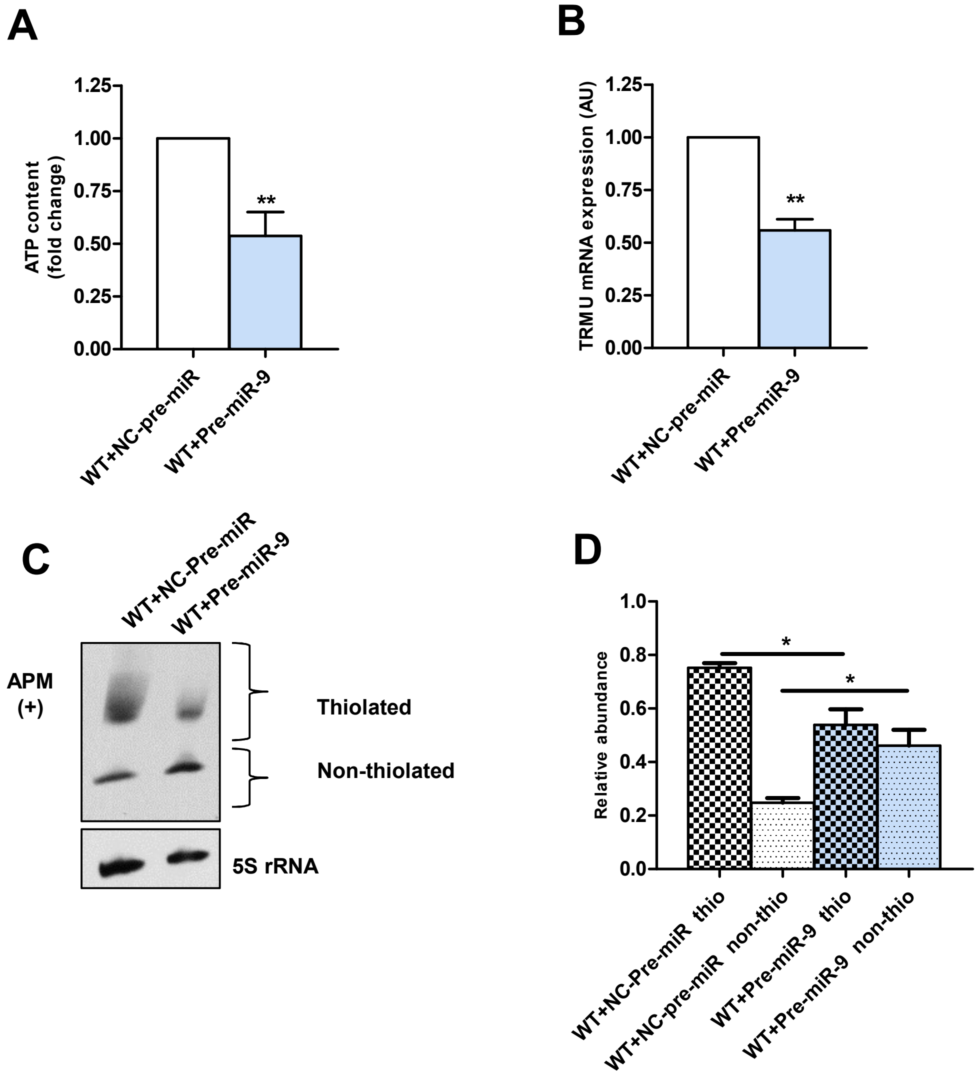

Figure S2. Analysis of the 2-thio-modified fraction of the mt-tRNA^{Lys} in cells transfected with pre-miRs. (A) Cellular ATP determination in pre-miR-9- and NC-pre-miR-transfected WT cells. **(B)** qRT-PCR analysis of *TRMU* mRNA expression in 143B cells (WT cybrids) transfected with either pre-miR-9 or the negative control (NC)-pre-miR. **(C)** APM-Northern analysis of the 2-thiolation status of mt-tRNA^{Lys} obtained from WT cells transfected with either pre-miR-9 or NC-pre-miR. The thiolated tRNAs were detected as retarded bands in the presence of APM. The membrane was also probed with 5S rRNA as a loading control. **(D)** Percentage of thiolated and nonthiolated mt-tRNALys species compared with the whole amount of this mt-tRNA. The quantification of each fraction (thiolated or nonthiolated) is expressed as a percentage of its signal from the total signal (thiolated + non-thiolated signals). All data represent the mean ± SD of at least three different experiments. Differences from NC values were found to be statistically significant at *p<0.05 and **p<0.01. AU: arbitrary units.

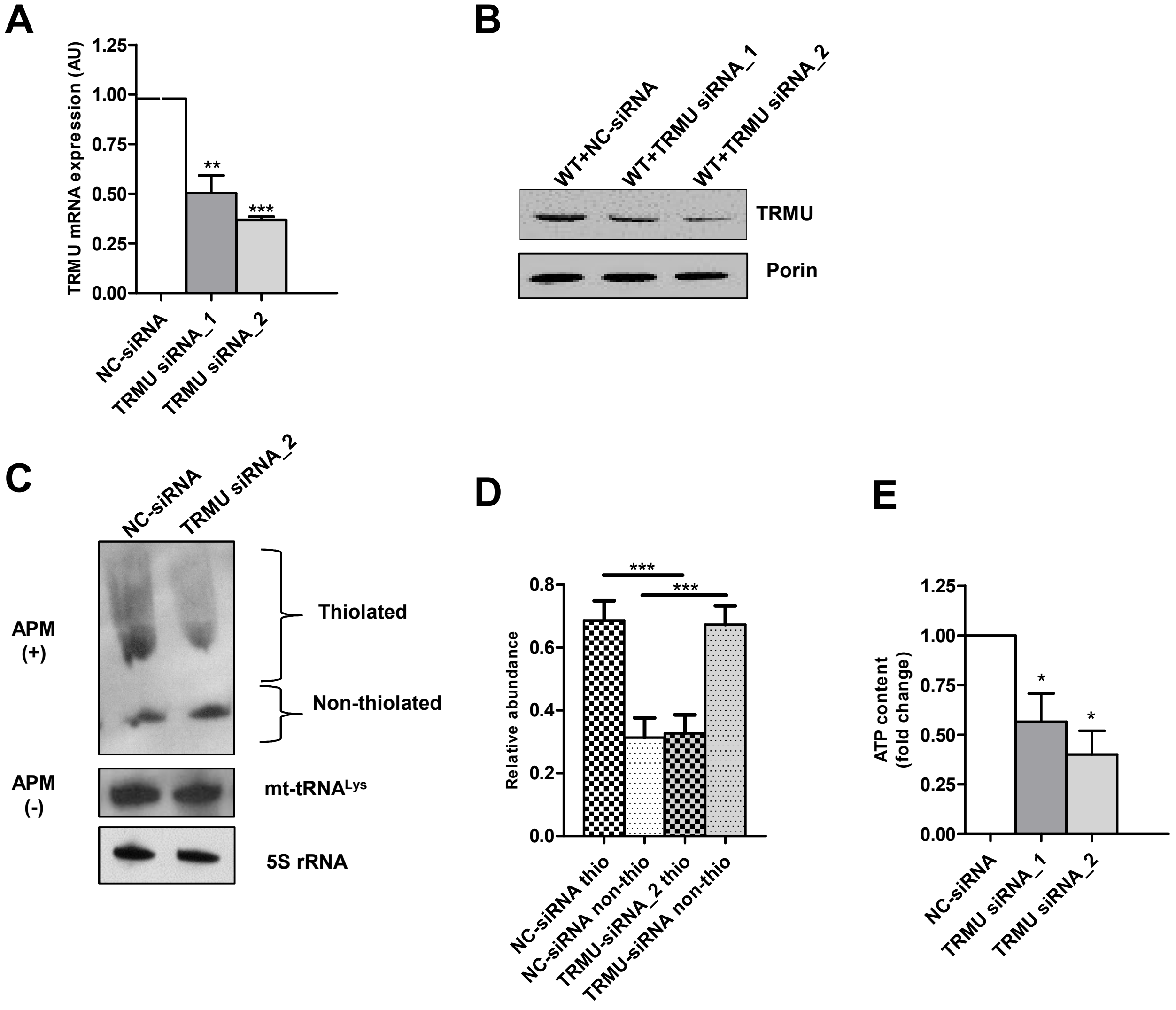

Figure S3. Transitory silencing of TRMU expression reduces ATP levels in 143B cells. (A) qRT-PCR analysis of *TRMU* mRNA expression in 143B cells (WT cybrids) transfected during 48h with specific TRMU siRNAs (TRMU siRNA_1 and _2) and the negative control (NC)-siRNA. **(B)** Representative western blot of TRMU in TRMU siRNA_1-, TRMU siRNA_2- and NC-siRNA- transfected WT cells. The membrane was also probed with porin as a loading control. **(C)** APM-Northern analysis of the 2-thiolation status of mt-tRNALys obtained from TRMU siRNA_2- and NC-siRNA- transfected WT cells. The same amount of total RNA (7,5 μg) was run in a denaturing polyacrylamide-urea gel in the presence (+) or absence (-) of APM. The thiolated tRNAs were detected as retarded bands in the presence of APM. The APM(-) membrane was also probed with 5S rRNA as a loading control. **(D)** Percentage of thiolated and nonthiolated mt-tRNALys species compared with the whole amount of this mt-tRNA. The quantification of each fraction (thiolated or nonthiolated) is expressed as a percentage of its signal from the total signal (thiolated + non-thiolated signals). **(E)** Cellular ATP determination in TRMU siRNA_1-, TRMU siRNA_2- and NC-siRNA- transfected WT cells. All data represent the mean ± SD of at least three different experiments. Differences from NC values were found to be statistically significant at *p<0.05, **p<0.01 and ***p<0.001. AU: arbitrary units.

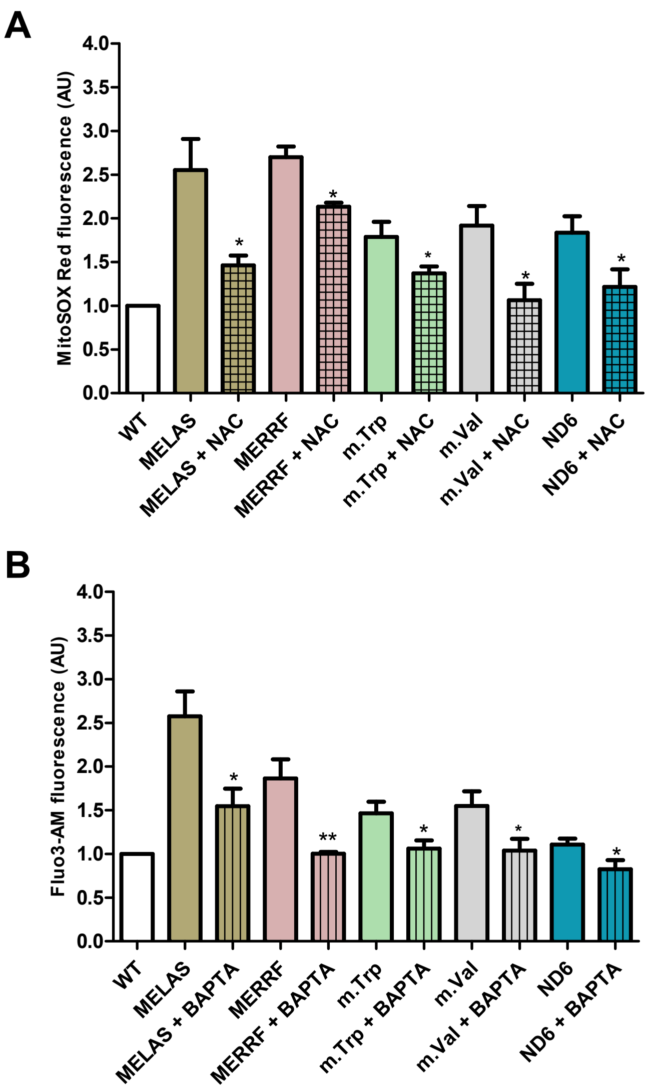

Figure S4. Reduction of ROS and intracellular Ca^{2+} levels in mutant and WT cybrid cells by treatment with NAC and BAPTA. (A) Determination of ROS by flow cytometry in mutant cybrid cells treated for 48h with 1 mM NAC using MitoSOX Red. **(B)** Determination of Ca^{2+} by flow cytometry in mutant cybrid cells treated for 2h with 10 mM BAPTA using Fluo3-AM. All data are the mean ± SEM of at least three different experiments. Differences from WT values were found to be statistically significant at $^{*}p<0.05$ and $^{**}p<0.01$. AU: arbitrary units.

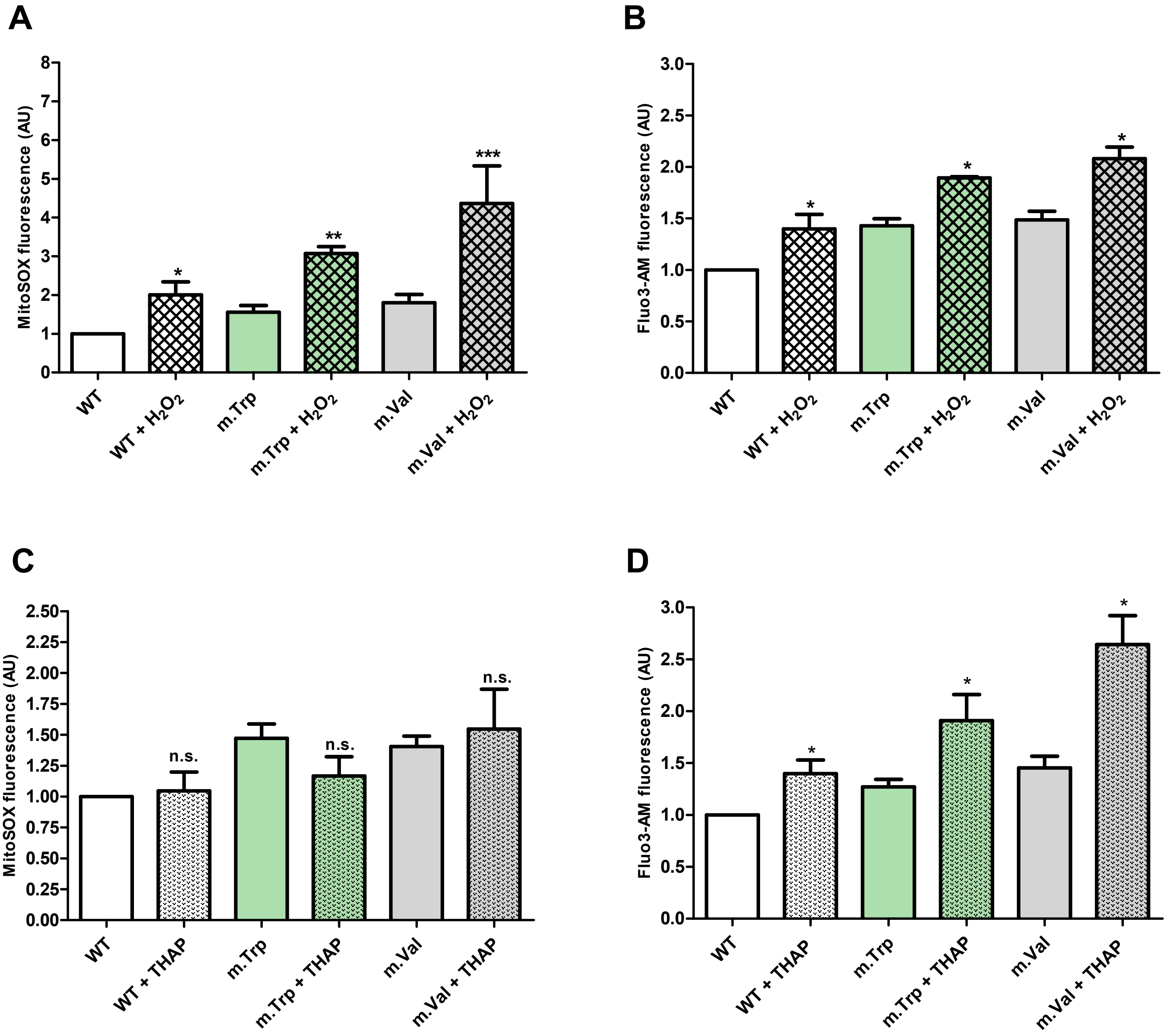

Figure S5. Treatment of m.Trp, m.Val and WT cybrid cells with H_2O_2 increases both ROS and intracellular Ca^{2+} levels, whereas Thapsigargin only elevates intracellular Ca^{2+}. (A and B) Determination of ROS (A) and intracellular Ca^{2+} (B) by flow cytometry in m.Trp, m.Val and WT cybrid cells treated with 5 mM H_2O_2 for 6h using MitoSOX Red and Fluo3-AM, respectively. **(C and D)** Determination of ROS (C) and intracellular Ca^{2+} (D) by flow cytometry in mutant cybrid cells treated with 400 nM Thapsigargin (THAP) for 24h using MitoSOX Red and Fluo3-AM, respectively. All data are the mean ± SEM of at least three different experiments. Differences from WT values were found to be statistically significant at $*p<0.05$, $**p<0.01$ and $***p<0.001$. AU: arbitrary units.

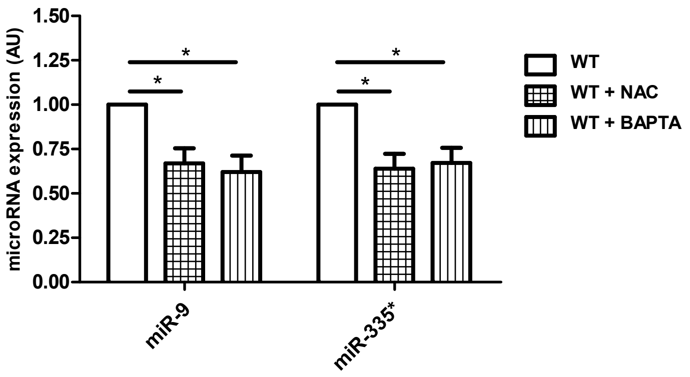

Figure S6. The wild type expression of miR-9 and miR-335* is affected by NAC and BAPTA treatments. qRT-PCR analysis of miR-9 and miR-335* expression in WT cybrid cells treated with either 10 µM BAPTA (Ca^{2+} chelator) for 2h or 1 mM NAC (antioxidant) for 48h. All data are the mean ± SEM of at least three different experiments. Differences from WT values were found to be statistically significant at *$p<0.05$. AU: arbitrary units.

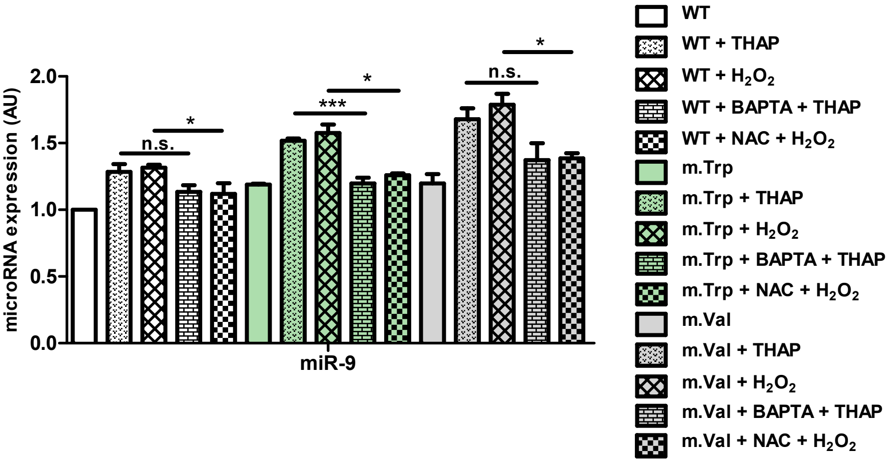

Figure S7. The induction of miR-9 in m.Trp and m.Val cells treated with hydrogen peroxide (H_2O_2) and Thapsigargin is due to the effects of the reagents on the levels of ROS and Ca^{2+}. qRT-PCR analysis of miR-9 expression in m.Trp, m.Val and WT cybrid cells treated with either 400 nM Thapsigargin (THAP) for 24h or 5 mM H_2O_2 for 6h, with or without a respective pretreatment with either 10 μM BAPTA (Ca^{2+} chelator) for 2h or 1 mM NAC (antioxidant) for 48h. All data are the mean ± SEM of at least three different experiments. Differences from WT values were found to be statistically significant at *$p<0.05$ and ***$p<0.001$. AU: arbitrary units.

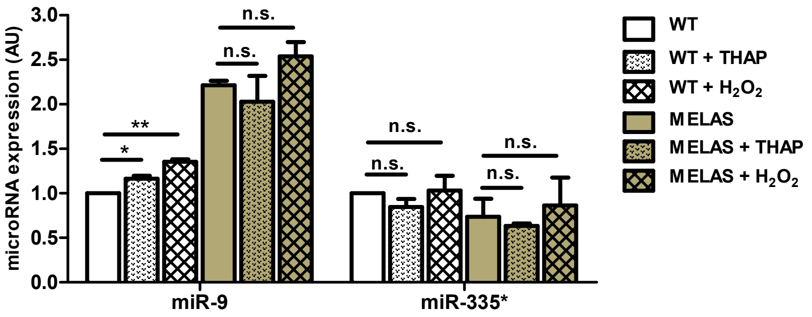

Figure S8. Expression of miR-9 is maximal in MELAS cybrids. qRT-PCR analysis of miR-9 and miR-335* expression in MELAS and WT cybrid cells treated with either 400 nM Thapsigargin (THAP) for 24h or 5mM H_2O_2 for 6h. Differences from WT values were found to be statistically significant at *$p<0.05$ and **$p<0.01$. AU: arbitrary units.

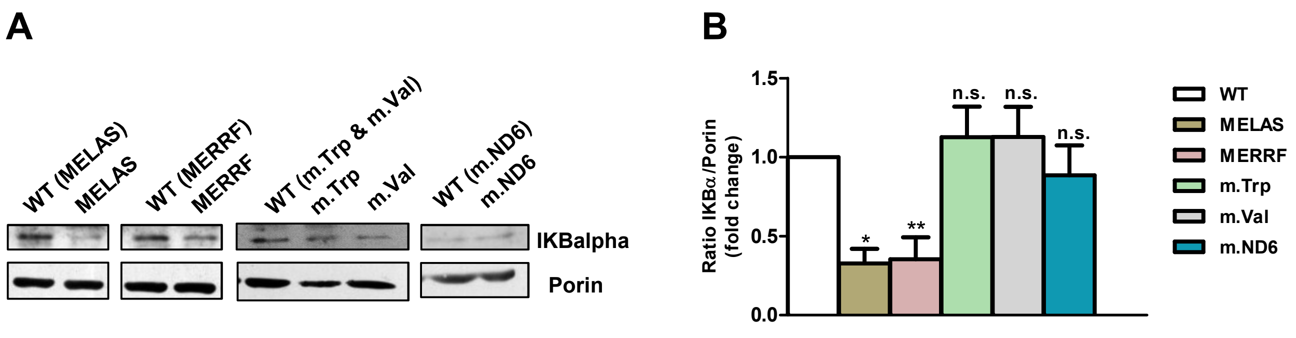

Figure S9. Expression of IKBα is compromised in MELAS and MERRF cybrids but not in m.Trp, m.Val, and m.ND6 cells. (A) Western blot analysis of IKBα in mutant and WT cybrid cells. The filters were also probed with porin as a loading control. **(B)** Densitometric analysis of IKBα normalized to the loading control and represented as fold change relative to WT. All data are the mean ± SEM of at least three different experiments. Differences from WT values were found to be statistically significant at $^*p<0.05$ and $^{**}p<0.01$.

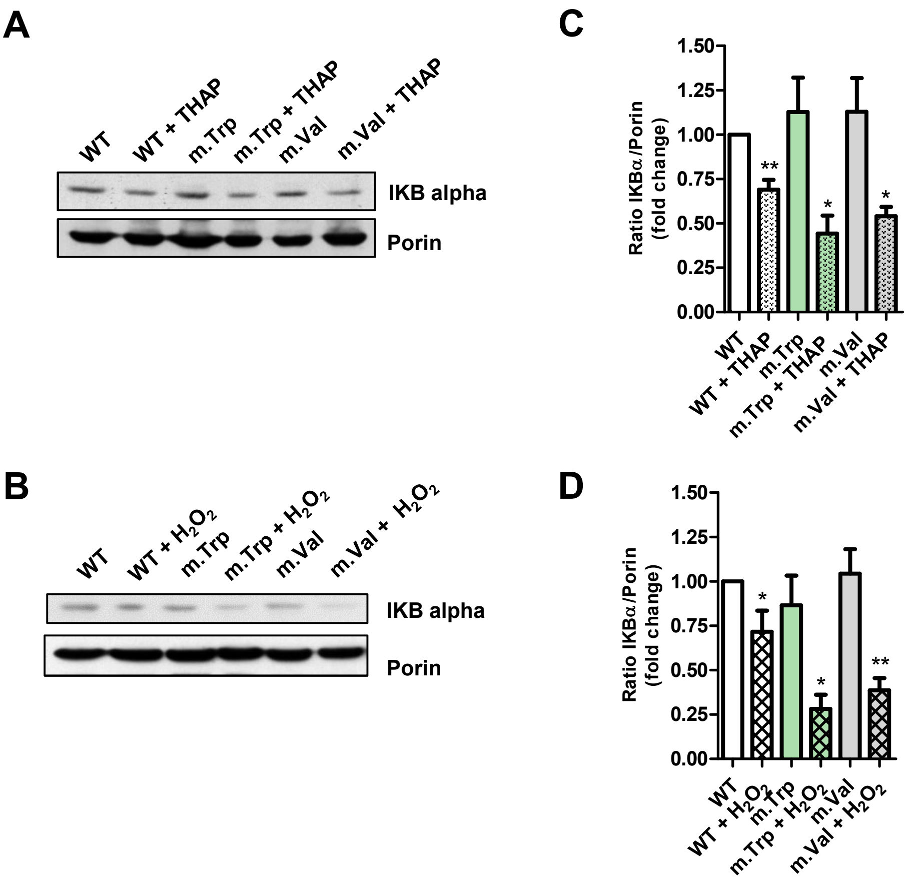

Figure S10. Treatment of m.Trp, m.Val and WT cybrid cells with either Thapsigargin or H_2O_2 reduces the IKBα expression. (A and B) Western blot analysis of IKBα in m.Trp, m.Val and WT cybrid cells treated with either 400 nM Thapsigargin (THAP) for 24h (A) or 5 mM H_2O_2 for 6h (B). The filters were also probed with porin as a loading control. **(C and D)** Densitometric analysis of IKBα normalized to the loading control and represented as fold change relative to WT. All data are the mean ± SEM of at least three different experiments. Differences from WT values were found to be statistically significant at *p<0.05 and **p<0.01.

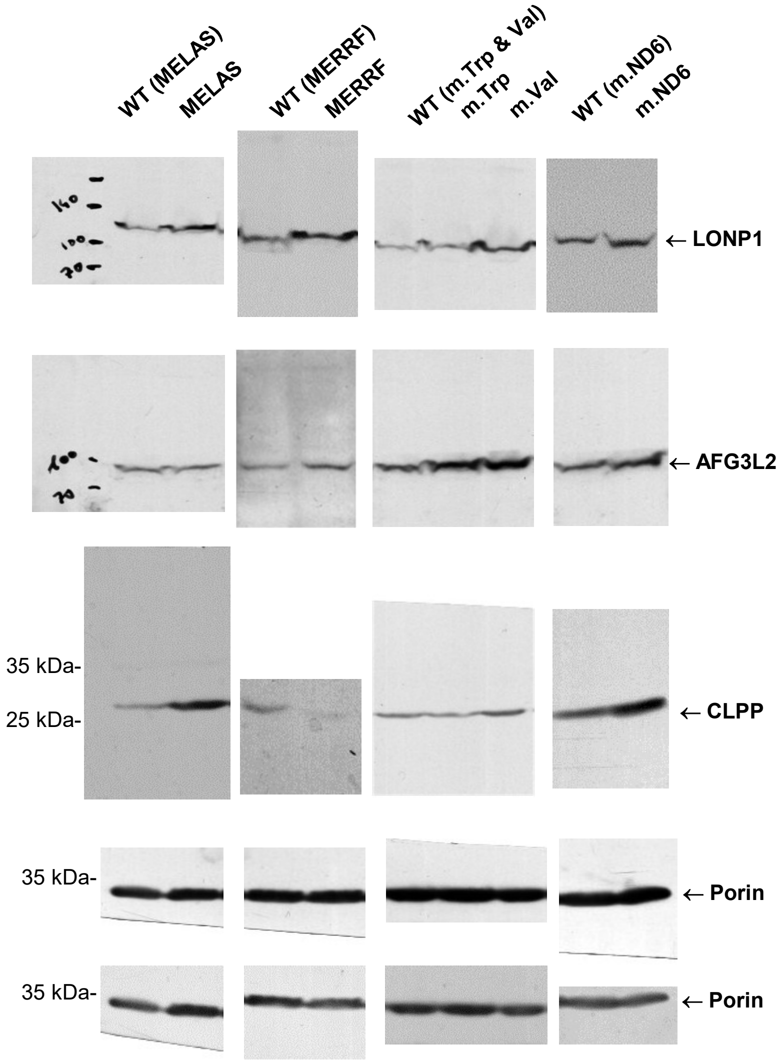

Figure S11. Full-length Western blots of LONP1, AFG3L2, CLPP and Porin in mutant and WT cybrid cells (shown in Fig. 1A). The full-length membrane was cutted and incubated with the respective antibody. Less exposed blots of Porin are shown at the bottom.

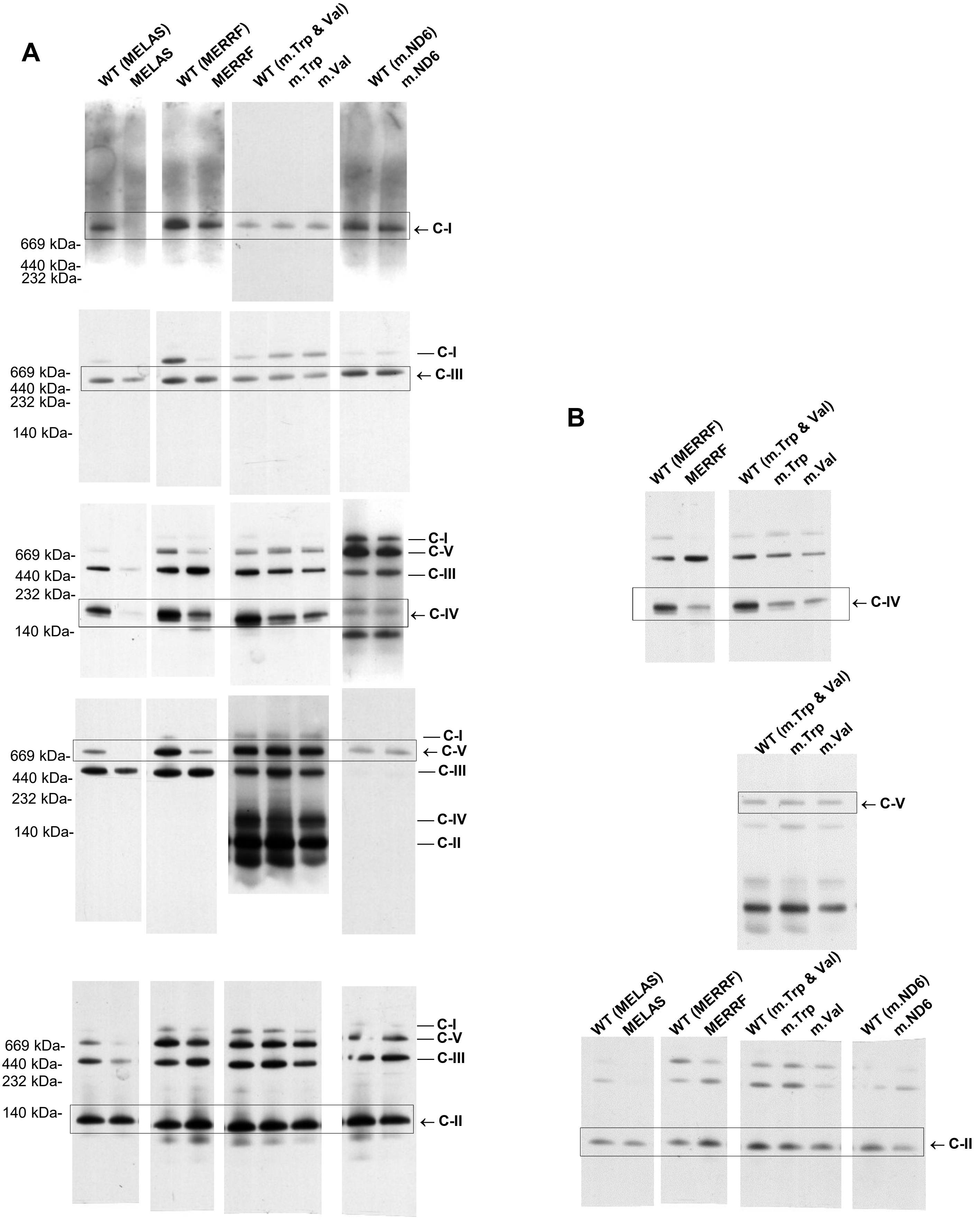

Figure S12. A. Full-length blots of OXPHOS complexes in mutant and WT cybrid cells (shown in Fig. 1C). Representative blots from independent experiments are shown. The presence of additional bands besides the expected one after the incubation with a specific antibody is due to previous antibody incubations. **B.** Less exposed blots are shown for those with high contrast in panel A.

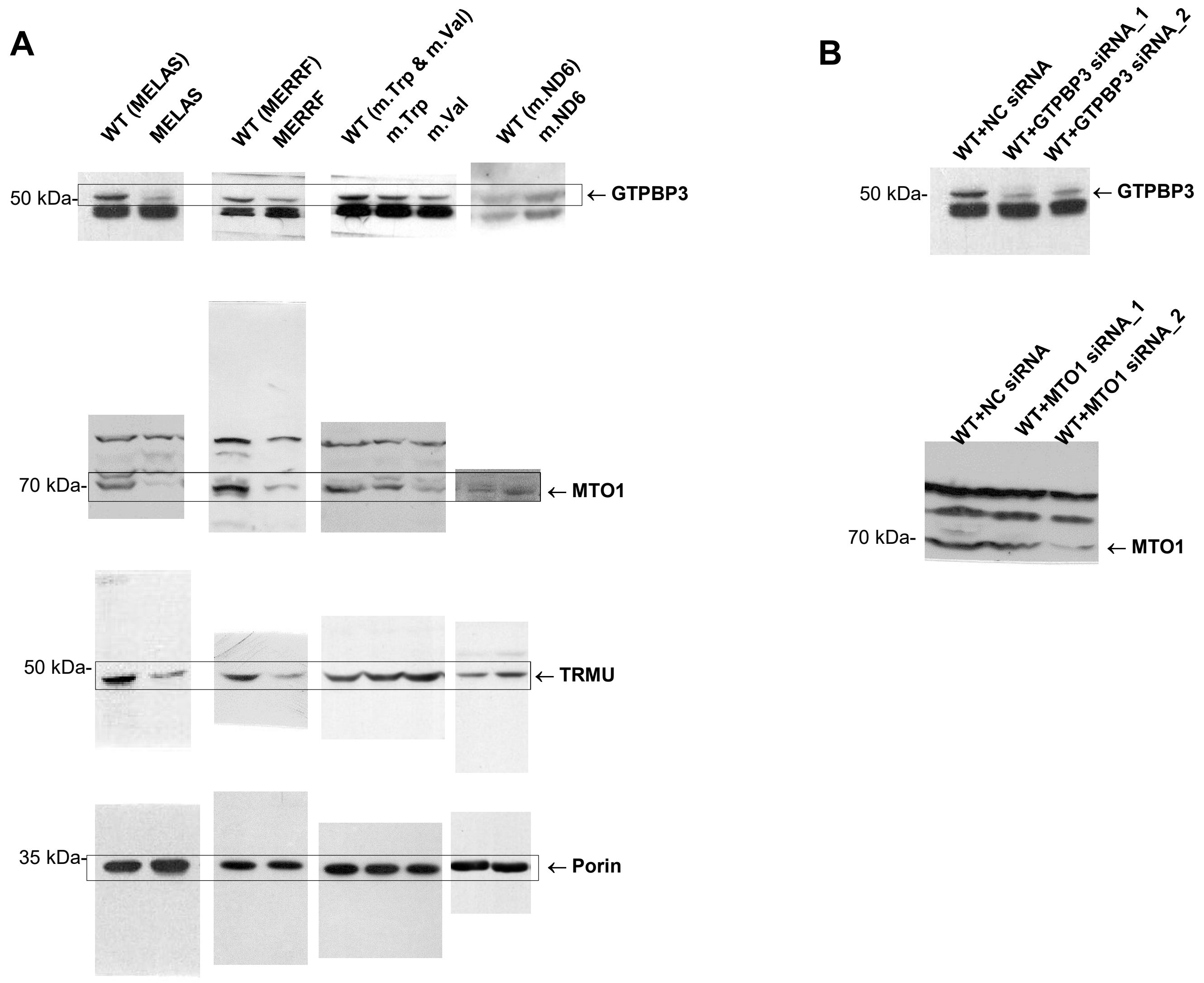

Figure S13. A. Full-length Western blots of GTPBP3, MTO1, TRMU and Porin in mutant and WT cybrid cells (shown in Fig. 2D). The full-length membrane was cutted and incubated with the respective antibody. **B.** Full-length Western blots of GTPBP3 (top) and MTO1 (bottom) in WT cybrid cells transfected with GTPBP3 and MTO1 siRNAs, respectively, showing that the antibodies anti-GTPBP3 and anti-MTO1 recognize a band of the expected size whose intensity is reduced after silencing.

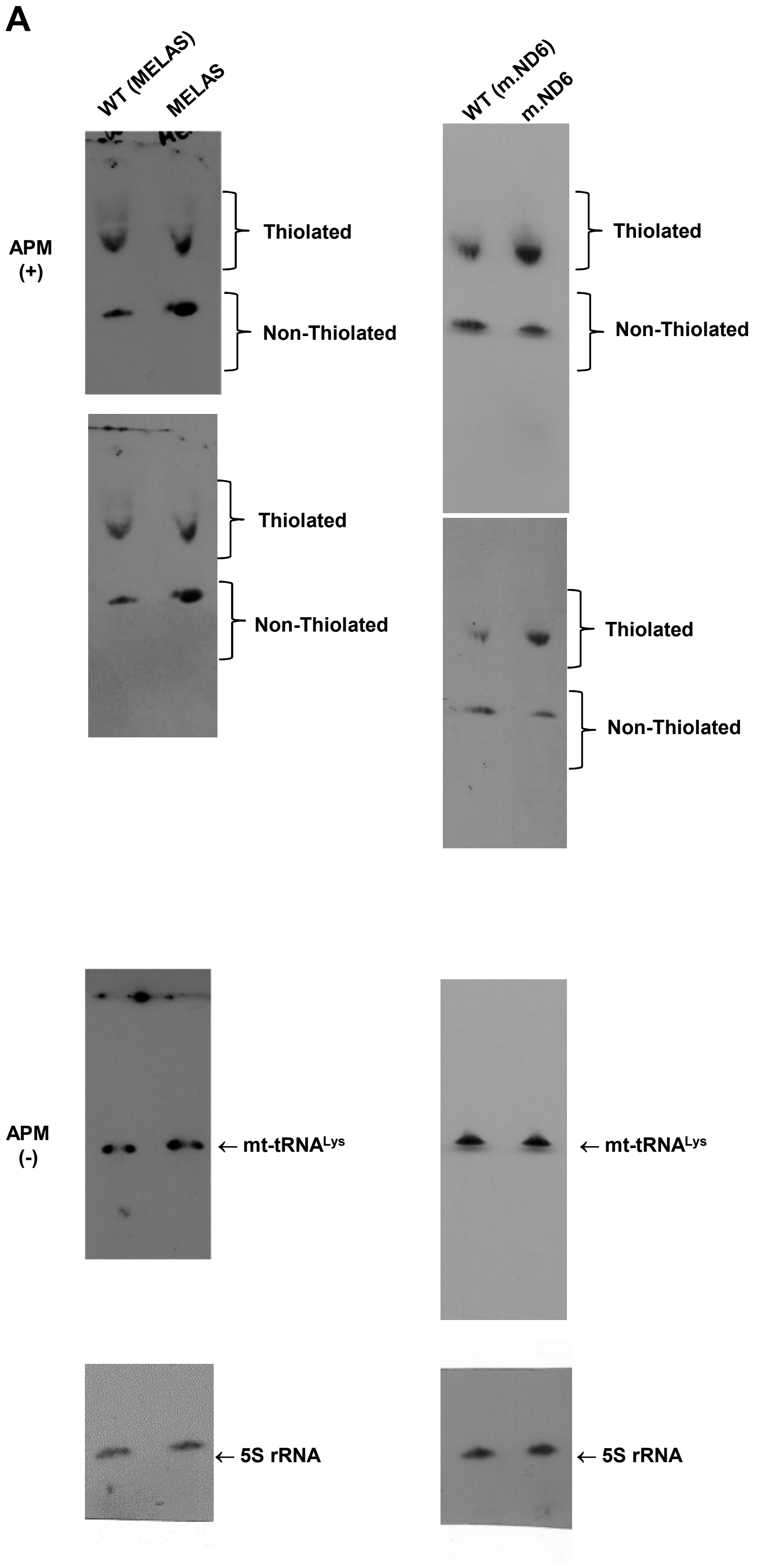

Figure S14. Full-length Northern blots of mt-tRNA^{lys} obtained from mutant and WT cybrid cells (shown in Fig. 3A). Less exposed blots are shown for the APM(+) membranes.

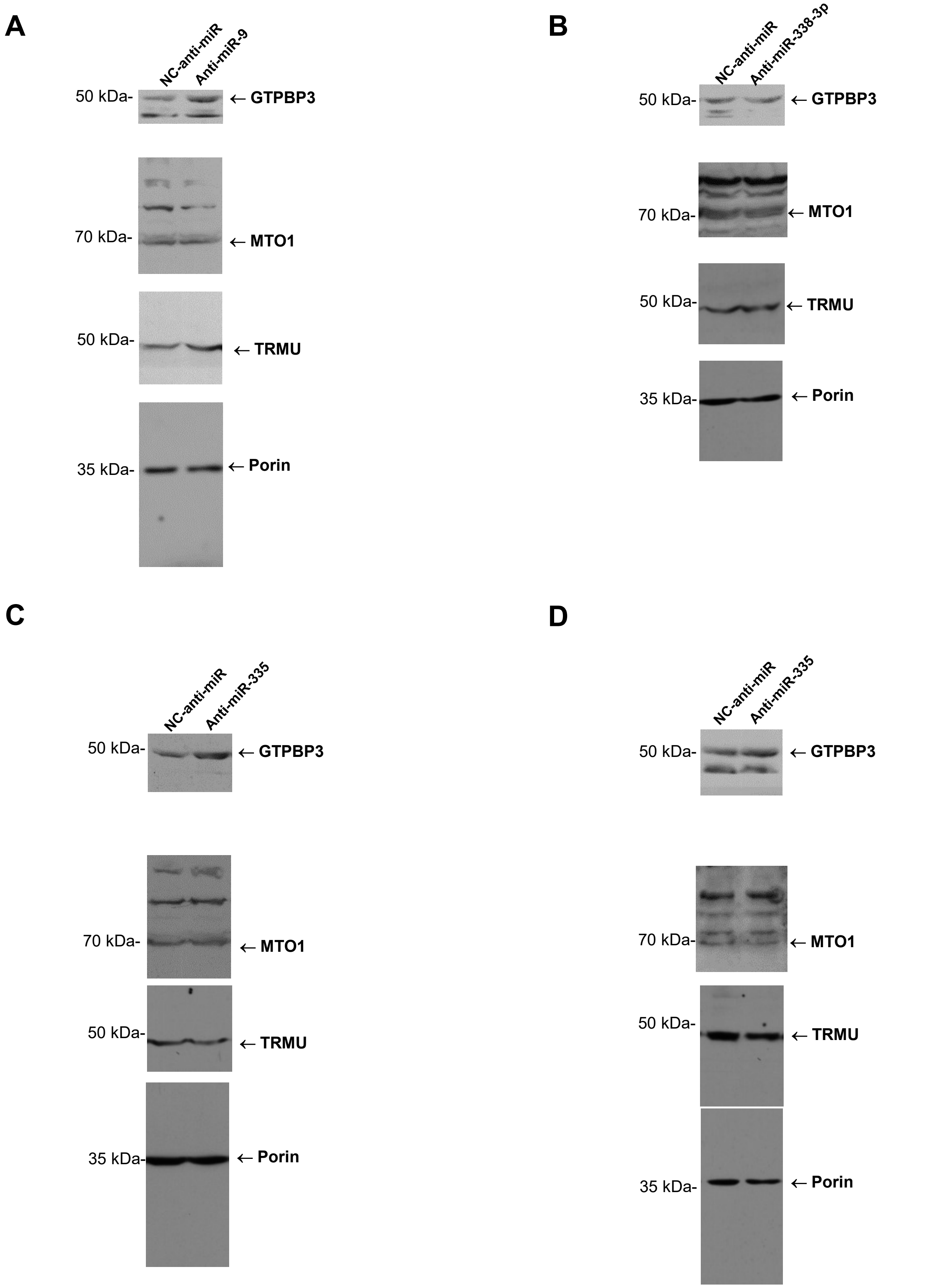

Figure S15. Full-length Western blots of GTPBP3, MTO1, TRMU and Porin in anti-miR-9-transfected MERRF cells (A), anti-miR-338-3p-transfected MELAS cells (B), anti-miR-335-transfected m.Trp cells (C) and anti-miR-335-transfected m.Val cells (D), and in the respective negative control (NC)-transfected cells (A-D) (shown in Fig. 5A-D). The full-length membrane was cutted and incubated with the respective antibody.

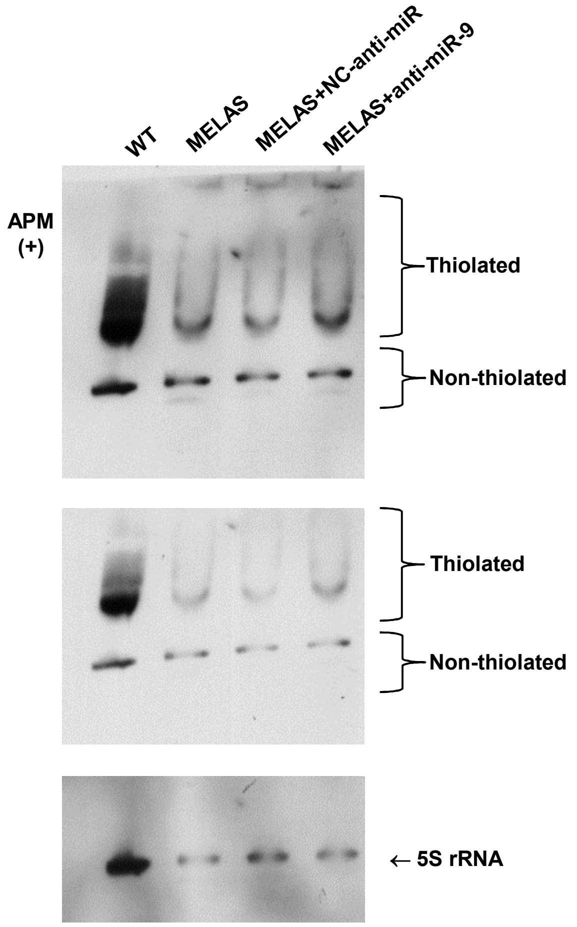

Figure S16. Full-length Northern blots of mt-tRNA^{lys} and 5S rRNA obtained from WT and MELAS cells transfected with either anti-miR-9 or NC-anti-miR (shown in Fig. 6F). A less exposed blot of mt-tRNA^{Lys} is shown at the bottom.

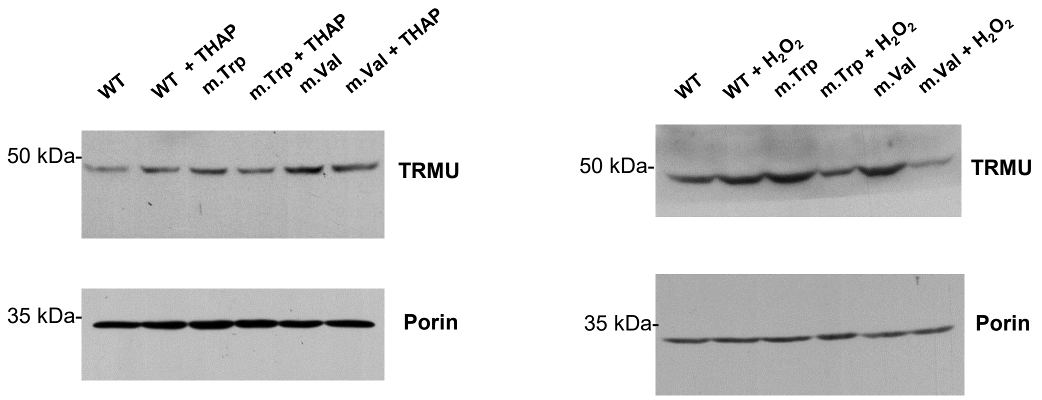

Figure S17. Full-length Western blots of TRMU and Porin in m.Trp, m.Val and WT cybrid cells treated with either 400 nM Thapsigargin (THAP) for 24h (left) or 5 mM H_2O_2 for 6h (right) (shown in Fig. 8C). The full-length membrane was cutted and incubated with the respective antibody.

	GTPBP3	TRMU	MTO1	SUM
**miR-9/-9***	3	1	2	6
**miR-335/-335***	3	0	2	5
**miR-141**	1	0	3	4
**miR-200a**	1	0	3	4
**miR-217**	1	0	1	2
**miR-26b**	1	0	1	2
**miR-338-3p**	3	3	0	6
**miR-15a/-15a***	1	3	0	4
**miR-15b/-15b***	1	3	0	4
**miR-34b/-34b***	3/2	0	0	5
**miR-125b-2***	*2*	0	0	2
**miR-30b***	*2*	0	0	2
**miR-210**	1	0	0	1
**miR-424**	0	3	0	3
**miR-16**	0	3	0	3
**miR-146a***	0	*1*	0	1
**miR-183/-183***	0	0	1/1	2
**miR-27a***	0	0	*2*	2
**miR-200c**	0	0	1	1
**miR-429**	0	0	1	1
**miR-199a**	0	0	1	1

Table S1. Predicted ROS-related miRNA binding sites in the 3'UTR of *GTPBP3, TRMU and MTO1* mRNAs. The total number of databases out of the three used (miRWalk, Targetscan and miRANDA) providing a miRNA binding site in the 3'UTR region of each mRNA (GTPBP3, TRMU, and MTO1) is indicated for each microRNA. Lack of a binding site is indicated by zero. miRNAs partially corresponding to the complementary strand of a miRNA are indicated by asterisks (*) and their respective hits in each mRNA are underlined. The list has been arranged in descending order of total hits (SUM, in grey) of the three genes. The common microRNA regulator for the three mRNAs is colored in pale red. Common microRNA regulators for *GTPBP3* and *MTO1* mRNAs are colored in pale green. Common microRNA regulators for *GTPBP3* and TRMU mRNAs are colored in orange. Specific microRNA regulators for *GTPBP3*, *MTO1* and *TRMU* mRNAs are colored in purple, dark green or pale blue, respectively.

Gene	Primer Name	Sequence (5' to 3')	Assay
GTPBP3	GTPBP3_F	RT² qPCR Primer Assay for Human GTPBP3 (QIAGEN)	qRT-PCR
	GTPBP3_R		
TRMU	TRMU_F	ggcttttcagaaatcggtttgaa	qRT-PCR
	TRMU_R	tgggaaacctggctgagaaaga	
MTO1	MTO1_F	cctgaaggaatggattctgac	qRT-PCR
	MTO1_R	gctgcagcttcctcataacc	
ACTB	ACTB_F	tgagcgcggctacagctt	qRT-PCR
	ACTB_R	tccttaatgtcacgcacgatt	
SDH	SDH_F	tctccagtggccaacagtgtt	mtDNA copy number quantification
	SDH_R	gccctcttgttcccatcaac	
COXII	COXII_F	cgatccctcccttaccatca	mtDNA copy number quantification
	COXII_R	ccgtagtcggtgtactcgtaggt	
mt-tRNALys	mt-tRNALys dig	tggtcactgtaaagaggtgttggt	Northern blot
5S rRNA	5S rRNA dig	gggtggtatggccgtagac	Northern blot

Table S2. List of oligonucleotides used in this work. "F" indicates forward primer" and "R" denotes reverse primer.
